# A Two-Step Strategy for the Rapid Enrichment of *Nitrosocosmicus*-Like Ammonia-Oxidizing Thaumarchaea

**DOI:** 10.3389/fmicb.2019.00875

**Published:** 2019-04-24

**Authors:** Liangting Liu, Surong Li, Jiamin Han, Weitie Lin, Jianfei Luo

**Affiliations:** Guangdong Provincial Key Laboratory of Fermentation and Enzyme Engineering, School of Biology and Biological Engineering, South China University of Technology, Guangzhou, China

**Keywords:** ammonia-oxidizing archaea, *Nitrosocosmicus*, biofilms, quartz sand, ciprofloxacin, azithromycin

## Abstract

Ammonia-oxidizing archaea (AOA) are widely distributed on the earth and play a significant role in the global nitrogen cycle. Although dozens of AOA strains were obtained in the last 13 years, it is still necessary to obtain more AOA strains for the entire exploration of their ecology, physiology, and underlying biochemistry in different environments. In this study, we designed a two-step strategy for the rapid enrichment of *Nitrosocosmicus*–like AOA from soils. Firstly, combination of kanamycin and ampicillin was chosen as the selective stress for bacteria and quartz sands were used as the attachment of AOA cells during the first step cultivation; only after 40–75 days cultivation, AOA enrichments with abundance >20% were obtained. Secondly, combination of ciprofloxacin and azithromycin was chosen as the selective stress for the following cultivation; it is able to penetrate the biofilms and kill the bacterial cells inside the aggregate, contributing to the AOA enrichments reached high abundances (90%) only after one-time cultivation. Basing on this strategy, three AOA strains were obtained from agricultural soils only after 90–150 days cultivation. Phylogenetic analysis suggested these AOA belong to the soil group I.1b *Thaumarchaeota* and are closely related to the genus *Nitrosocosmicus*. In general, AOA enrichment or isolation is very difficult and time-consuming (an average of 2–3 years). Here, we provide a new strategy for the rapid enrichment of high abundance of *Nitrosocosmicus*-like AOA from soil, which gives a new solution to the AOA enrichment and cultivation in a short period.

## Introduction

Autotrophic aerobic ammonia oxidation is the primary step in oxidizing ammonia to nitrate and is therefore central to the global nitrogen cycle (Kowalchuk and Stephen, 2001). This biochemical reaction could be performed by ammonia-oxidizing bacteria (AOB), ammonia-oxidizing archaea (AOA), and comammox bacteria (Beeckman et al., 2018). However, for a long time, AOB were assumed to be the sole drivers of ammonia oxidation in the environment. It was not until 2005, when the first AOA strain, *Nitrosopumilus maritimus* SCM1, was successfully isolated, that the member of AOA was recognized as one of the contributors to ammonia oxidation (Könneke et al., 2005). From then on, detection, enrichment and cultivation of AOA belonging to the phylum Thaumarchaeota have been widely carried out (Francis et al., 2007; Lehtovirta-Morley, 2018). Autotrophic ammonia-oxidizing microorganisms all possess ammonia monooxygenase (AMO), but the overall stoichiometry of it in AOA is indistinguishable from that of AOB and shows a higher affinity to ammonia (Martens-Habbena et al., 2009; Kits et al., 2017; Kuypers, 2017). AOA appear to be adapted to life under nutrient limitation (Horak et al., 2013; Shiozaki et al., 2016), which suggests that they have a significantly broader habitat range than the characterized AOB. They appear to be the dominant archaeal clade in soil (generally comprising 1–5% of all prokaryotes) (Ochsenreiter et al., 2003; Lehtovirta et al., 2009; Tago et al., 2015), the marine system (comprising 20–40% of all marine bacterioplankton) (Karner et al., 2001; Church et al., 2003), and geothermal habitats (Zhang et al., 2008; Dodsworth et al., 2011).

According to their performances on the ammonia oxidation in most natural systems, AOA have been believed to play a significant role in the global nitrogen cycle (Leininger et al., 2006; Pratscher et al., 2011; He et al., 2012). However, their roles have not been studied as extensively as AOB; it is still necessary to fully explore their ecology, physiology, and underlying biochemistry in environments (Stahl and de la Torre, 2012). Then, it is urgent to obtain more AOA isolates or enrichments. Up to now, 32 different AOA strains distributing in eight archaeal genera (*Nitrosopumilus, Nitrosocaldus*, *Nitrosopelagicus*, *Nitrososphaera*, *Nitrosotalea*, *Nitrosoarchaeum*, *Nitrosotenuis*, and *Nitrosocosmicus*) have been reported (Supplementary Table S1). Though dozens of AOA isolates and enrichments were obtained in the last 13 years, a time-saving strategy of AOA enrichment and isolation has rarely been reported in this field.

Due to the low maximum specific growth rate (0.011–0.033 h^-1^) and inhibition by low concentrations of ammonia (2–100 mM) and nitrite (0.028–5.7 mM) (Lehtovirta-Morley et al., 2016), it usually takes a very long time to obtain AOA enrichment from natural samples. Although some organics (such as pyruvate, oxaloacetate, malate, etc.) were reported to be able to promote the growth of AOA (Tourna et al., 2011; Kim et al., 2016; Sauder et al., 2017), they were also consumed by the symbiotic heterotrophic bacteria in the enrichment cultures. More importantly, AOA members related to the *Nitrosocosmicus* clade can produce extracellular polymeric substances (EPS) to form cell aggregates or biofilms, which provides nutrition and protection for bacterial cells (Flemming et al., 2016; Jung et al., 2016; Kerou et al., 2016). Antibiotics (such as Streptomycin, Kanamycin, and Ampicillin) are often used as the selective stress for the AOA enrichment and purification (Supplementary Table S1). However, the application of antibiotics often stimulates the biofilm formation and the bacterial antibiotic resistance (Hoffman et al., 2005; Kaplan, 2011).

**Figure 1:**
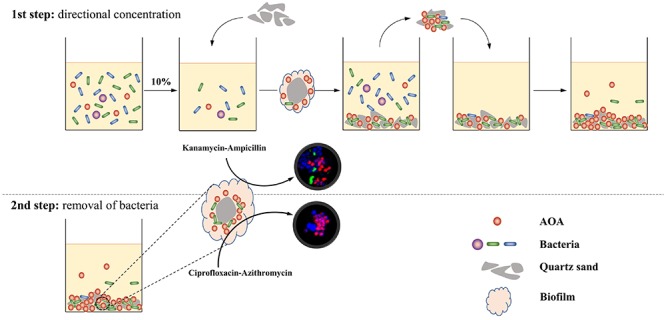
Schematic process of a strategy for rapid enrichment of high abundance AOA. During the first step, the AOA cells were directly absorbed by quartz sands and concentrated on the surface of quartz sands; during the second step, the antibiotic combination ciprofloxacin-azithromycin penetrates biofilms and kills bacteria in the aggregate.

In this study, we designed a two-step strategy for the rapid enrichment of AOA from the environment (Figure 1). During the first step, soil samples were cultivated in the culture media containing no antibiotic; after the identification of nitrite in the culture, 10% of the initial enrichment was transferred into the subculture using kanamycin-Ampicillin as selective stress for the bacterial growth, and quartz sands as attachment for the AOA cells; in the following transfer, the quartz sands in each subculture were obtained and used as inoculums for the next subculture. During the second step, the quartz sands were collected when AOA abundance on the attachment reached 20% and were transferred into a new subculture using Ciprofloxacin-Azithromycin as selective stress; after 2 to 3 subcultures, high abundance of AOA enrichment could be obtained. Using this strategy, three AOA enrichments (abundance >90%) that closely related to the genus *Nitrosocosmicus* were obtained from agricultural soils, after only 90 to 150 days of cultivation.

## Materials and Methods

### Characterization of Soil Sample

Soil samples were collected from paddy fields, garden and vegetable fields (Supplementary Table S2). The diversity of AOA distributed in these soils were studied using high-throughput sequencing archaeal 16S rRNA and *amoA* genes, which generated an average of 9,109 and 8,838 filtered reads, respectively (Supplementary Table S3). Bioinformatics analysis indicated that the AOA community in these soils mainly consisted of *Nitrosocosmicus*, *Nitrososphaera*, *Nitrosopumilus*, *Nitrosotenuis*, and *Nitrosotalea* (Supplementary Figure S1); the Shannon estimator from alpha diversity indices indicated that the SS (Suishi village) soil had the highest AOA diversity and abundance of AOA (Supplementary Table S3). Based on these results, the SS soil was used as an environmental sample for the AOA enrichment in this study.

### Cultivation and Enrichment

Five grams of soil collected from the SS site were inoculated into 100 mL of the culture medium and initialized the AOA enrichment in accordance with the two-step strategy. Cultivation of ammonia oxidizer was carried out using an mineral salts medium containing NaCl (1 g L^-1^), MgCl_2_⋅6H_2_O (0.4 g L^-1^), CaCl_2_⋅2H_2_O (0.1 g L^-1^), KH_2_PO_4_ (0.2 g L^-1^), KCl (0.5 g L^-1^), and filtration-sterilized solutions including 1 mL L^-1^ trace element solution (1.5 g L^-1^ FeCl_2_⋅4H_2_O, 190 mg L^-1^ CoCl_2_⋅6H_2_O, 100 mg L^-1^ MnCl_2_⋅6H_2_O, 70 mg L^-1^ ZnCl_2_, 62 mg L^-1^ HBO_3_, 36 mg L^-1^ Na_2_MoO_4_⋅2H_2_O, 24 mg L^-1^ NiCl_2_⋅6H_2_O, and 17 mg L^-1^ CuCl_2_⋅2H_2_O), 1 mL L^-1^ Fe-EDTA solution (details are presented in the Supplemental Materials), 336 mg L^-1^ NaHCO_3_, and 107 mg L^-1^ NH_4_Cl; the pH value of medium was adjusted to 7.0. Each enrichment culture was incubated at 30°C in the dark without shaking and transferred into fresh media by the time the ammonium concentration was reduced to about 20% of the initial level. 50 mg L^-1^ of streptomycin, kanamycin, ampicillin, carbenicillin, and tetracycline in different combinations were supplied to the mineral salt medium. Based on the accumulation of nitrite, an effective antibiotic combination was chosen as the selective stress for subcultures. During the cultivation, the concentrations of nitrite and ammonium were determined by Griess-Ilosvay method and indophenol blue method, respectively (ISO/TS 14256–1:2003, 2003). In order to remove fungi contamination, 10 mg L^-1^ natamycin was added to the initial culture medium. During the first transfer, the cultures were filtered through a 5 μm filter to remove soil debris and the bacterial cells embedded in the massive biofilms.

To assess the effect of quartz sands on the directional concentration of AOA, 10% (v/v) of enrichment cultures were transferred into the fresh liquid media containing 2, 5 or 10% (w/v) of quartz sands (∼ 1 mm diameter). After the ammonium was reduced to 20% of the initial level, the liquid cultures and quartz sands were collected and used for the AOA quantitative analysis, respectively. After the assessment, media containing 10% (w/v) of quartz sands were chosen for the following subcultures. During each transfer, all of the quartz sands in the culture were collected and transferred into fresh media for the next subculture. When the ammonia-oxidizing rate (nitrite-producing rate) and archaea abundance in subcultures reach a stable phase (the relative abundance of AOA in enrichment has no increase), antibiotics including kanamycin, ampicillin, tobramycin, ciprofloxacin, azithromycin, tetracycline, polymyxin, lincomycin, and spiramycin were used to evaluate the effect on the removal of bacterial cells in the biofilm that formed during the AOA enrichment.

### Genomic DNA Extraction

To extract the total DNA in liquid culture, 20 mL enrichment culture was collected and filtered through a cellulose filter (0.22 μm, Thermo Fisher Scientific); the filter retaining with cells was collected and cut into pieces and placed in a 2 mL grinding tube containing 1 g quartz sand. To extract the total DNA from the quartz sand, 1 g quartz sand from the enrichment culture was collected and placed in a 2 mL grinding tube. 0.5 mL CTAB extraction buffer (10% CTAB, 0.7 M NaCl, 240 mM potassium phosphate buffer, pH 8.0) and 0.5 mL phenol-chloroform-isoamyl alcohol solution (25:24:1, pH 8.0) was added into the tube and mixed for 5 min using the Vortex Adapter (13000-V1-24, QIAGEN, Germany). After the pretreatment, genomic DNA was extracted according to a protocol that was previously reported by Griffiths (Griffiths et al., 2000). After the DNA extraction, 20 μg glycogen (Thermo Scientific, United States) was used as co-precipitant to deposit DNA from the DNA precipitated solution (30% polyethylene glycol 6000, 1.6 M NaCl). The DNA purity was determined using a UV spectrophotometer (NanoDrop 2000, Thermo Scientific, United States) and by agarose gel electrophoresis. The DNA samples were stored at -20°C for further PCR amplification.

### Gene Clone, Identification and Phylogenetic Analysis

Nearly complete 16S rRNA gene and archaeal *amoA* gene of the AOA enrichments were PCR amplified using primer pairs A21f/1492r and CrenamoA23f/CrenamoA616r (Supplementary Table S4), respectively. PCR products were purified, ligated into pMD^®^19-T Vector (Takara, Dalian, China), and transformed into *Escherichia coli* DH5α. Recombinant clones were picked and sequenced with RV-M and M13-47 vector specific primers by IGE Biotechnology (Guangzhou, China).

Evolutionary histories of archaeal 16S rRNA gene nucleotide sequences and *amoA* gene translated protein sequences were inferred using the maximum likelihood method, based on the Tamura 3-parameter model. All alignments and phylogenetic analyses were conducted by the software MEGA 7 (Kumar et al., 2016).

### Quantitative PCR

Archaeal and bacterial 16S rRNA genes were PCR amplified using primer pairs SS16S-1F/SS16S-1R (a primer set specific for *Ca.* Nitrosocosmicus sp., designed with primer-blast tool in NCBI, according to the 16S rRNA gene sequences from genus *Nitrosocosmicus*) and 1369F/1492R (Supplementary Table S4), respectively. Agarose gel purified PCR products of archaeal or bacterial 16S rRNA genes were cloned using the TA-cloning kit (Takara, Dalian, China). Plasmids were extracted using the TIANpure Mini Plasmid Kit II (TIANGEN, Beijing, China) and digested using QuickCut^TM^ Hind III (Takara, Dalian, China). The linearized plasmid was used to construct the standard curves. Standard curves were prepared using six serial tenfold dilutions ranging from 10^2^ to 10^7^ gene copies/mL. The DNA was quantified by determining the copy number as well as the concentration and base pair composition of related genes. All quantitative PCR were performed in triplicate on an ABI 7500 Fast real-time PCR system (Applied Biosystems) using TransStart Tip Green qPCR SuperMix (Transgen, Beijing, China). The reaction condition was as follows: 2 min at 94°C; 40 cycles of 10 s at 94°C and 34 s at 60°C. The correlation coefficients (*R*^2^) of the standard curves were 0.999. The amplification efficiencies (*E*) of archaeal and bacterial 16S rRNA gene were 85 to 93%.

### Microscopy

To observe the biofilms formed on the surface of quartz sands, glass slides were used as the attachment and laid vertically in the enrichment cultures. After incubation, the slides were directly subjected to the Fluorescence *in situ* hybridization (FISH) analysis. FISH was performed by a modified method as previously described by Nielsen et al. (2009). In brief, samples were fixed with 50% ethanol (mixed with PBS) at 30°C for 48 h. After resuspending in PBS, samples were immobilized on Poly (L-lysine) slides and dehydrated by graded ethanol (50%, 80% and 98%, each gradient was treated for 3 min); cells were incubated with proteinase K (50 ug/mL) for 30 min at 37°C and washed three times using dH_2_O. After permeabilized, cells were hybridized at 46°C for 3 h in hybridization buffer with 35% formamide using an Alexa Fluor 488 labeled probe Eub338 and an Alexa Fluor 546 labeled probe Arch915 (Supplementary Table S4). After hybridization, samples were covered with prewarmed washing buffer and incubated at 48 °C for 20 min, followed by covering with ProLong^TM^ Diamond Antifade Mountant with DAPI (Invitrogen, United States). Microscopic observation and documentation were accomplished using a scanning confocal microscope (LSM 710, Carl Zeiss, Germany) and the ZEN 2011 black software.

For scanning electron microscopy (SEM), the fixed cells were mounted on an aluminum stub and sputter-coated with platinum using a sputter coater EMS150T (EMS, United Kingdom), then imaged using a FEI Q25 scanning electron microscope (FEI, United States).

### Nucleotide Sequence Accession Number

The nucleotide sequences accession number of the cultured AOA (16S rRNA and *amoA* gene sequence for phylogenetic analysis) and soil sample (amplicon raw data for community analysis) are summarized in Supplementary Table S5.

## Results

### Cultivation and Enrichment of AOA

To find a suitable antibiotic for suppressing bacterial growth at the beginning of AOA enrichment, the nitrite that produced in ammonia oxidation was used as the evaluation index for antibiotic selection. As results show in Supplementary Figure S2, addition of kanamycin and ampicillin as the selective stress had 2.44 mg L^-1^ nitrite accumulation, which was higher than the other antibiotic combinations, indicating that they could be used for the AOA enrichment.

**Figure 2:**
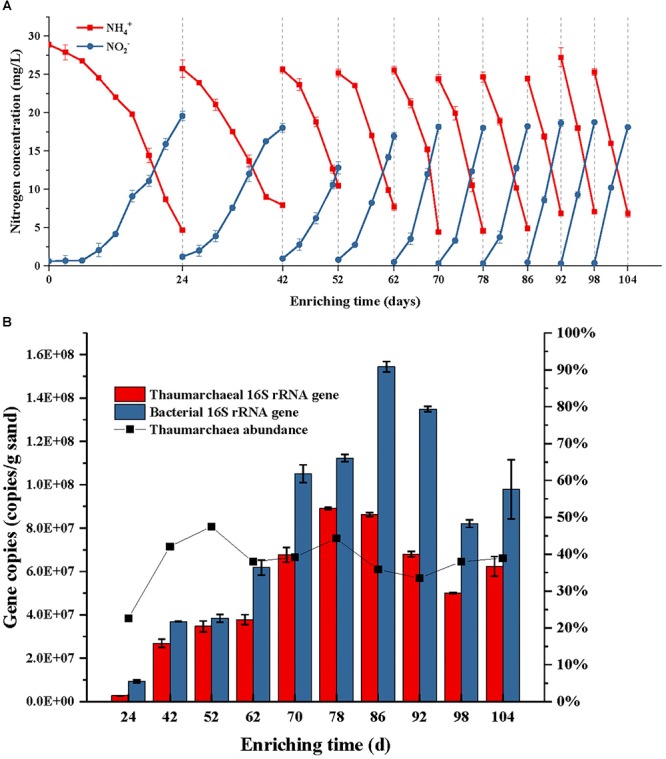
Ammonia consumption and nitrite accumulation **(A)**, as well as thaumarchaeal and bacterial 16S rRNA gene copies **(B)** of the AOA enrichment during each subculture in use of kanamycin and ampicillin as selective stress; the ammonium with a concentration of 28 mg/L NH4^+^-N was added into the initial culture medium and each subculture; when the concentration of NH4^+^-N was reduced to about 20% of the initial level, the sands in the enrichment were obtained and transferred into a fresh medium for subculture. Error bars indicate the standard error of the mean for biological triplicates.

During the first subculture in the first step, the NH_4_^+^ consumption contributed to about 19.57 mg L^-1^ NO_2_^-^-N production after 24 days cultivation (Figure 2A). The PCR detection performed using the bacterial *amoA* primers 1F/2R, suggested the absence of AOB in the first step enrichment. Since then, the period time of enrichment cultivation was shortened to 10 days in the third subculture, 8 days in the fifth subculture, and 6 days in the eighth subculture. From the eighth subculture, the ammonia-oxidizing rate and nitrite-producing rate always remained at stable levels, which indicated that the AOA enrichment cultivation reached a stable phase. This result was consistent with the result of a quantitative PCR analysis, that the archaea abundance in the cultures dynamically changed between 34 and 39% after the fourth subculture (Figure 2B). In fact, the archaea abundance had already reached 47% after the third subculture; however, the abundance no longer continuously increased after that time, while only archaeal and bacterial cells increased simultaneously at the same time (Figure 2B). This result suggests that the presences of some bacterial species in the enrichment culture obtained a tolerance or resistance to the activities of kanamycin and ampicillin and then survived under the antibiotic selective stress.

**Figure 3:**
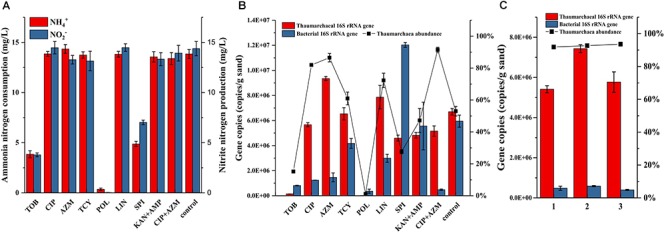
Ammonia consumption and nitrite accumulation **(A)**, as well as thaumarchaeal and bacterial 16S rRNA gene copies **(B)** in the AOA enrichments using new antibiotics as selective stress; TOB: tobramycin, CIP: ciprofloxacin, AZM: azithromycin, TCY: tetracycline, POL: polymyxin, LIN: lincomycin, SPI: spiramycin, 50 mg/L of each antibiotic was added before inoculation. Thaumarchaeal and bacterial 16S rRNA gene copies, and archaea abundance in the AOA enrichments of three times repetition of the enrichment using ciprofloxacin and azithromycin as selective stress **(C)**. Error bars indicate the standard error of the mean for biological triplicates.

The resistance of bacteria to antibiotics might come from the protection by biofilms that formed by the AOA growing on the surface of quartz sands. Some AOA strains belonging to the *Nitrosocosmicus* clade have been reported to form aggregates and biofilms (Jung et al., 2016). Similarly, biofilms were also observed to form on the surface of quartz sands (Supplementary Figures S3a,b). Even though the biofilms were not as thick as the biofilms that were formed by many heterotrophic bacteria, they may still have the potential to decrease the activities of antibiotics. To inhibit the biofilm formation or suppress the bacterial growth in the biofilm, some new antibiotics were applied and assessed for their effects. As results shown in Figure 3, addition of tobramycin or polymyxin inhibited the ammonia oxidation of AOA enrichments, as well as the archaeal and bacterial growth in the enrichment cultures; the spiramycin not only inhibited the ammonia oxidation, but also resulted in a large increase of bacteria; tetracycline and lincomycin in some extent had no influence on the ammonia oxidation and showed positive effects on limiting the bacterial growth, which contributed to 61–72% of archaea abundance in the enrichment cultures. In comparison with other antibiotics, ciprofloxacin and azithromycin have no influence on the ammonia oxidation and contributed to the archaea abundances as high as 82% and 87%, respectively (Figure 3B). Moreover, the combination of ciprofloxacin and azithromycin resulted in 91% of the archaea abundance, which was about two times higher than that used in the combination of kanamycin and ampicillin. In the following, subcultures using ciprofloxacin-azithromycin, the archaea abundances remained steady at high levels ranging from 92 to 94% (Figure 3C).

During the AOA cultivation, the bacterial and archaeal cells would aggregate together and form biofilms in the absence of antibiotic (Supplementary Figure S3c). With the presence of kanamycin and ampicillin, thicker biofilms were formed, and more bacteria grew inside the biofilms (Supplementary Figure S3d). Differently, the combination of ciprofloxacin and azithromycin was able to limit the formation of thick biofilms (Supplementary Figure S3e). The comparison of FISH observation indicated that the combination was able to penetrate the biofilms and kill most of the bacterial cells (Supplementary Figures S3c–e). The stable high-abundance of AOA in the enrichment cultures indicated that the addition of ciprofloxacin combined with azithromycin as a selective stress is effective in the removal of bacteria in the aggregate, obtaining high abundance of AOA from the environment in a short-period.

### Characterization of High Abundance AOA Enrichment

**Figure 4:**
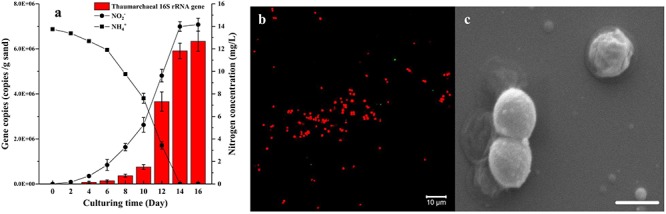
Growth curve **(a)**, FISH profile **(b),** and SEM micrograph **(c)** of AOA enrichment culture (AOA abundance >95%) growing in the culture medium containing 14 mg/L NH4^+^-N and using ciprofloxacin and azithromycin as selective stress. On the FISH profile, archaeal 16S rRNA was labeled with Alexa Fluor 546 Archaea-specific probe (Arc915, red) and bacterial 16S rRNA was labeled with Alexa Fluor 488 labeled Bacteria-specific probe (EUB338, green), objective: Plan-Apochromat 40×/1.3 Oil DIC M27; SEM condition was HV = 5.00 kV, mag = 40000×, scale bar = 1 μm. Error bars indicate the standard error of the mean for biological triplicates.

Based on the two-step strategy, one enrichment with high abundance of AOA was obtained after 150 days of cultivation. This AOA enrichment was able to consume 13.74 mg L^-1^ NH_4_^+^-N and reached a cell density of 6.34 × 10^6^ cells g^-1^ sand in 14 days (Figure 4a). The FISH profile indicated that only a few bacterial cells was detected in the enrichment culture (Figure 4b), which is consistent with the result of quantitative PCR analysis. The SEM micrograph indicated that these archaeal cells are coccoid and 0.6–1.2 μm in diameter (Figure 4c). They often appeared in groups or aggregation, which may be covered in an extracellular matrix. On the basis of archaeal 16S rRNA and *amoA* gene sequences, the archaea strain in the enrichment culture belongs to the soil group I.1b *Thaumarchaeota*, specifically in the *Nitrosocosmicus* cluster, and is closely related to *Ca.* Nitrosocosmicus exaquare G61, showing 99.6% and 94.3% similarity, respectively. According to the phylogenetic analysis, the Thaumarchaea is named as *Ca.* Nitrosocosmicus sp. SS.

### Application of the Two-Step Strategy to Other Soils

**Figure 5:**
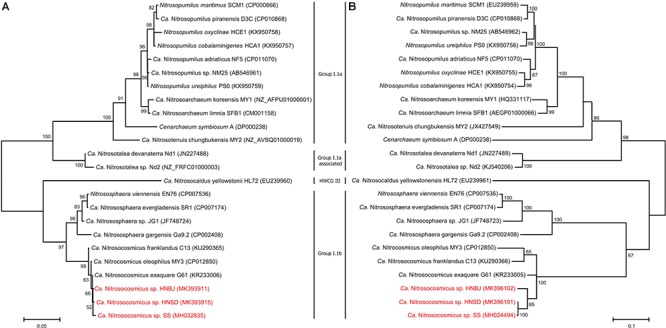
Phylogenetic analyses of AOA 16S rRNA gene **(A)** and amoA gene **(B)** by using Maximum Likelihood method. Bootstrap values over 50% based on 1000 replicates are shown.

Soil samples that were collected from paddy fields (HN_SD) and banana fields (HN_BJ) were subjected to the AOA enrichment using the methods described above. The newly collected soils were checked for the presence of AOA species using PCR amplification of the *amoA* gene (Supplementary Figure S4a). During the first-step cultivation, the AOA abundance in the enrichments of HN_SD and HN_BJ soils reached 20% only after 75 days of cultivation (Supplementary Figures S4b–d), similar to the previous enrichment. During the second step, the AOA abundance in the enrichments of HN_SD and HN_BJ soils reached 91% and 89%, respectively, after only 12 days of cultivation (Supplementary Figure S4d). The FISH profiles also indicated that only a small number of bacterial cells were detected in the two enrichment cultures (Supplementary Figures S4e,f). Phylogenetic analysis, based on archaeal 16S rRNA and *amoA* gene sequences, suggested that the newly obtained archaea in the two enrichments are also affiliated with the genus *Nitrosocosmicus*, named *Ca.* Nitrosocosmicus sp. HNSD and *Ca.* Nitrosocosmicus sp. HNBJ, respectively (Figure 5).

## Discussion

In the last two decades, 32 AOA pure cultures and enrichments were obtained from different environments (Supplementary Table S1). However, the vast majority of AOA in natural ecosystems is uncultured and thus the ecology, physiology, and biochemistry of this vital N-cycling clade is still largely unknown, especially in soils. In this study, a two-step strategy was proposed and used for the rapid enrichment of AOA from soils. During the first step, the AOA cells were directly concentrated on the surface of quartz sands by the formation of biofilms. However, the bacterial cells were also embedded in the biofilms (Supplementary Figure S3a), which contributed to a low abundance of AOA in the enrichments. Then, in the second step, an antibiotic combination ciprofloxacin-azithromycin was applied to penetrate the biofilms and kill the bacterial cells inside the aggregate. Based on the strategy, high abundance AOA enrichments were obtained from agricultural soils after only 90 to 150 days of cultivation. This period is much shorter than other published AOA strains, like the hot spring groups *Ca.* Nitrosotenuis uzonensis N4 (7 years), *Ca.* Nitrososphaera gargensis (6 years) and *Ca.* Nitrosocaldus cavascurensis (4 years), the soil groups *Ca.* Nitrosotenuis chungbukensis MY2 (3 years), *Ca.* Nitrosotalea sp. Nd2 (3 years) and *Nitrososphaera viennensis* EN76 (2 years), and the manmade ecosystem groups *Ca.* Nitrosocosmicus exaquare G61 (3 years) and *Ca.* Nitrosotenuis cloacae SAT1 (1 year) (Supplementary Table S1).

**Figure 6:**
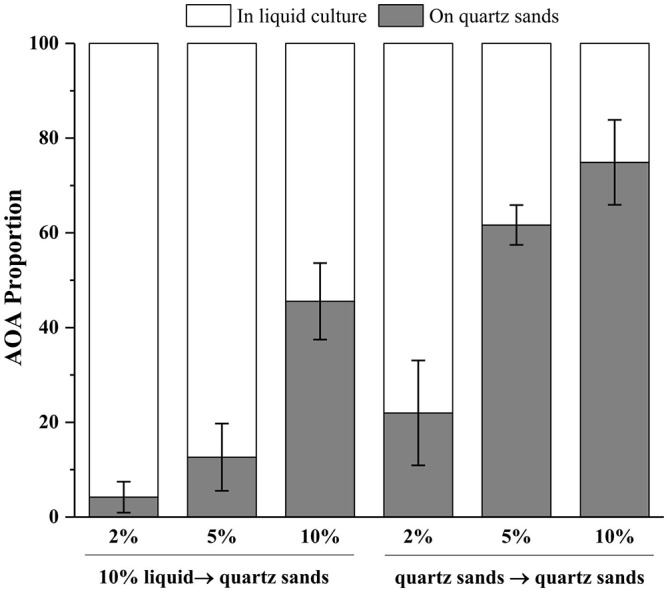
Proportions of AOA in the liquid cultures and on the quartz sands. 10% liquid → quartz sands means 10% enrichment culture was transferred into the fresh media containing 2, 5, or 10% (w/v) quartz sands; quartz sands → quartz sands means all of the quartz sands in the enrichment cultures containing 2, 5, and 10% quartz sands were collected and transferred into the fresh media. Error bars indicate the standard error of the mean for biological triplicates.

In an oligotrophic environment, the CO_2_ fixing activity of chemolithoautotrophic microorganisms often represents the primary organic source of complex microbial communities (Dattagupta et al., 2009; Denef et al., 2010). Similarly, during cultivation in inorganic media, AOA is the sole primary producers in the system and thus the sole source for organic carbon, which supports growth of heterotrophic bacteria and indirectly initiates the cells aggregate and biofilm formation on the surface of quartz sands. Notably, the member of the *Nitrosocosmicus* clade has usually been reported to form aggregates and biofilms by the secretion of EPS (Jung et al., 2016; Lehtovirta-Morley et al., 2016). It was reported that 77% and 73% of the cells of *Ca.* Nitrosocosmicus sp. MY3 were attached to hydrophobic bead and vermiculite, respectively; no significant attachment was observed for *Ca.* Nitrosotenuis chungbukensis (Jung et al., 2016). In addition, the genomic analysis indicated that members of Nitrososphaerales (such as *Nitrososphaera viennensis*) encode an extensive repertoire for biofilm formation including EPS production and cell surface modification (Kerou et al., 2016). Although the potential of biofilm formation has not been extensively studied in AOA, these results indicate that the presence of solid attachments (such as quartz sands) might benefit the rapid enrichment of AOA from environmental samples via the directional concentration of biofilms. As results show in Figure 6, the more quartz sands used the more the AOA attached on the quartz sands after 30 days of cultivation; with the addition of 10% (w/v) quartz sands, 46% of the AOA in enrichment culture attached on the surface of quartz sands when the culture inoculated with 10% of liquid seed, while 75% of the AOA attached on the quartz sands when the culture inoculated with quartz sands (attached with AOA cells). Moreover, the addition of kanamycin and ampicillin promoted the formation of biofilms, which might result in the concentration of AOA to the surface of quartz sands in a short time (Figure 4b).

During the enrichment cultivation, the AOA and bacterial cells formed a stable aggregate in biofilms, especially in the presence of antibiotics (Supplementary Figures S3a,b,d). Biofilm-induced antibiotic resistance has widely been reported in pathogenic bacteria (Hoffman et al., 2005); however, it is seldomly reported or discussed in AOA cultivation. Though it has become clear that archaeal biofilms are ubiquitous in a natural environment; we are still, however, far from comprehending the molecular mechanisms of interaction among the bacteria and the archaea in biofilm based antibiotic pressure (Orell et al., 2013). It is suggested that the presence of a bacterial partner in the biofilm stimulates AOA growth and enhances aggregate formation (Jung et al., 2016). The EPS synthesized by AOA could be used by heterotrophic bacteria as a substrate and embed the bacterial cells in the biofilm, which contributes to bacterial antibiotic resistance (Hallstoodley et al., 2004; Flemming et al., 2016). Moreover, the bacterial partners can reduce reactive oxygen stress of AOA by scavenging H_2_O_2_. This is beneficial for AOA, since they produce H_2_O_2_ during their growth, but many species lack a catalase for detoxification of the produced H_2_O_2_. Recently, it was shown that the absence of bacterial partners or H_2_O_2_ scavenging α-keto acids resulted in the inhibition of AOA growth (Kim et al., 2016). The H_2_O_2_ detoxification by bacterial partners harboring catalases is a key mechanism for supporting the AOA growth and would be a reason why it is difficult to remove the bacteria during the AOA enrichment. However, in this study, addition of different concentrations of pyruvate had no significant effect on improving the ammonia oxidation (Supplementary Figure S5). Catalase genes have been reported in the genomes of some soil AOA, such as *Nitrososphaera viennensis* EN76 (Stieglmeier et al., 2014), *Ca*. Nitrosocosmicus oleophilus MY3 (Jung et al., 2016) and *Ca*. Nitrosocosmicus exaquare (Sauder et al., 2017), the produced H_2_O_2_ could be scavenged by the catalases that produced by themselves, suggesting their growth independent of bacterial H_2_O_2_ scavengers. Overall, the detection of catalase genes in *Nitrosocosmicus* species and the unaffected ammonia oxidation with added pyruvate as H_2_O_2_ scavenger indicates that H_2_O_2_ detoxification might play only a minor role in the interaction of bacteria and *Nitrosocosmicus*-like AOA enrichments.

As discussed above, the interaction between AOA and bacterial partners in the enrichment culture allows them to better adapt to environmental stresses (such as antibiotic). To obtain highly enriched or even pure AOA cultures, this bacterial-archaeal interaction in the biofilm must be halted. In clinical therapy, some antibiotics (such as ciprofloxacin and azithromycin) have been proven to be available when killing pathogenic bacteria by destroying their biofilms (Saini et al., 2015). Azithromycin could slow biofilm formation down, penetrate the biofilms and reduce antibiotic resistance of bacterium as a quorum sensing inhibitor (Gillis and Iglewski, 2004 Persson et al., 2005; Yamamoto et al., 2015); Ciprofloxacin is a broad-spectrum fluoroquinolone antibiotic with good bactericidal activity and can prevent biofilm formation and reduce the preexisting biofilms (Reffuveille et al., 2014). Accordingly, we applied these antibiotics to the AOA enrichment. In comparison with the biofilms that formed in the cultures without addition of antibiotics and with addition of kanamycin and ampicillin, the biofilms in the culture with ciprofloxacin and azithromycin, appeared small and thin (Supplementary Figures S3d,e), which probably proves that the presence of these antibiotics can slow the formation of biofilms down. Higher hybridization rates of FISH also indicated that the presence of ciprofloxacin and azithromycin could increase the permeability of biofilms. Moreover, the dispersed cell that grows without addition of quartz sand but with addition of antibiotics were applied to further explore the potential reason why the combination of ciprofloxacin and azithromycin has a positive effect on improving FISH hybridization. As results show in Supplementary Figure S3, the hybridization rate of *Ca*. Nitrosocosmicus sp. SS, that cultivated under ciprofloxacin and azithromycin (Supplementary Figure S3f), were higher than that under kanamycin and ampicillin (Supplementary Figure S3g). Ichimiya et al. (1996) had found that the azithromycin was able to inhibit the production of extracellular polysaccharides. It seemed that if fewer EPS coated on the cell surface, more fluorescent probes entered the cells. In summary, the addition of ciprofloxacin combined with azithromycin as selective stress in the cultivation effectively inhibited cell aggregation and biofilms formation, resulting in the removal of most cells and thus to high abundance of AOA in the cultures. Chen et al. (2017) have used ciprofloxacin and/or azithromycin in combination with streptomycin, kanamycin, ampicillin, and tetracycline to cultivate AOA enrichment. Unfortunately, the growth of AOA was significantly reduced or totally inhibited by using the antibiotic combinations. The presence of ciprofloxacin and azithromycin inhibit the cell aggregation and biofilm formation, which are believed to be essential for the AOA survival in the environment, suggesting that these antibiotics cannot be used in initial AOA enrichment. By contrast, in the second step of the AOA enrichment strategy in this study, biofilms with a relatively high abundance of AOA were formed after the first step of concentration by quartz sands; the addition of ciprofloxacin-azithromycin penetrated the biofilms and killed the bacterial cells inside the aggregate, but had no influence on the AOA growth.

It is tempting to speculate that biofilm-forming, terrestrial AOA species, such as members of genera *Nitrososphaera*, *Nitrosotenuis*, and *Nitrosotalea*, can also be enriched using this two-step enrichment strategy. Many AOA species had been detected in the soil samples; however, only the member of *Nitrosocosmicus* was obtained after the enrichment in this study. It is suggested that this member forms biofilms faster under antibiotic stress and attaches to quartz more easily or grows faster under ciprofloxacin and azithromycin.

## Conclusion

In conclusion, the low specific growth rate, bacterial-archaeal interaction, and some other unknown features during the AOA enrichment leads to a lengthy of period time, before obtaining abundance of AOA from the environment. The two-step strategy discussed in this study, that addition of quartz sands to directly concentrate AOA cells and application of ciprofloxacin-azithromycin to effectively remove the bacterial cells inside biofilms, is an effective and timesaving method to obtain high abundance of *Nitrosocosmicus*–like AOA from the environment. To some extent, with appropriate adjustments, this strategy could be suitable for the enrichment of other AOA members in soils.

## Author Contributions

JL, LL, and WL conceived and designed the experiments. LL, JH, and SL performed the experiments and analyzed the data. LL, JL, and WL wrote the manuscript. All authors reviewed, edited, and approved the manuscript.

## Conflict of Interest Statement

The authors declare that the research was conducted in the absence of any commercial or financial relationships that could be construed as a potential conflict of interest.

## References

[B1] Beeckman F. Motte H. Beeckman T. (2018). Nitrification in agricultural soils : impact, actors and mitigation. *Curr. Opin. Biotechnol.* 50 166–173. 10.1016/j.copbio.2018.01.014 29414056

[B2] Chen H. Yue Y. Jin W. Zhou X. Wang Q. Gao S. (2017). Enrichment and characteristics of ammonia-oxidizing archaea in wastewater treatment process. *Chem. Eng. J.* 323 465–472. 10.1016/j.cej.2017.04.130

[B3] Church M. J. DeLong E. F. Ducklow H. W. Karner M. Preston C. M. Karl D. M. (2003). Abundance and distribution of planktonic archaea and bacteria in the waters west of the antarctic peninsula. *Limnol. Oceanogr.* 48 1893–1902. 10.4319/lo.2003.48.5.1893

[B4] Dattagupta S. Schaperdoth I. Montanari A. Mariani S. Kita N. T. Valley J. W. (2009). A novel symbiosis between chemoautotrophic bacteria and a freshwater cave amphipod. *ISME J.* 3 935–943. 10.1038/ismej.2009.34 19360027

[B5] Denef V. J. Mueller R. S. Banfield J. F. (2010). AMD biofilms: using model communities to study microbial evolution and ecological complexity in nature. *ISME J.* 4 599–610. 10.1038/ismej.2009.158 20164865

[B6] Dodsworth J. A. Hungate B. A. Hedlund B. P. (2011). Ammonia oxidation, denitrification and dissimilatory nitrate reduction to ammonium in two US Great Basin hot springs with abundant ammonia-oxidizing archaea. *Environ. Microbiol.* 13 2371–2386. 10.1111/j.1462-2920.2011.02508.x 21631688

[B7] Flemming H. C. Wingender J. Szewzyk U. Steinberg P. Rice S. A. Kjelleberg S. (2016). Biofilms: an emergent form of bacterial life. *Nat. Rev. Microbiol.* 14 563–575. 10.1038/nrmicro.2016.94 27510863

[B8] Francis C. A. Beman J. M. Kuypers M. M. (2007). New processes and players in the nitrogen cycle: the microbial ecology of anaerobic and archaeal ammonia oxidation. *ISME J.* 1 19–27. 10.1038/ismej.2007.8 18043610

[B9] Gillis R. J. Iglewski B. H. (2004). Azithromycin Retards *Pseudomonas aeruginosa* Biofilm Formation. *J. Clin. Microbiol.* 42 5842–5845. 10.1128/jcm.42.12.5842-5845.2004 15583321 PMC535287

[B10] Griffiths R. I. Whiteley A. S. Anthony G. Donnell O. Bailey M. J. Donnell A. G. O. (2000). Rapid method for coextraction of DNA and RNA from natural environments for analysis of ribosomal DNA- and rRNA-based microbial community composition. *Appl. Environ. Microbiol.* 66 5488–5491. 10.1128/aem.66.12.5488-5491.2000 11097934 PMC92488

[B11] Hallstoodley L. Costerton J. W. Stoodley P. (2004). Bacterial biofilms: from the natural environment to infectious diseases. *Nat. Rev. Microbiol.* 2 95–108. 10.1038/nrmicro821 15040259

[B12] He J. Hu H. Zhang L. (2012). Current insights into the autotrophic thaumarchaeal ammonia oxidation in acidic soils. *Soil Biol. Biochem.* 55 146–154. 10.1016/j.soilbio.2012.06.006

[B13] Hoffman L. R. D’Argenio D. A. MacCoss M. A. Zhang Z. Jones R. A. Miller S. I. (2005). Aminoglycoside antibiotics induce bacterial biofilm formation. *Nature* 436 1171–1175. 10.1038/nature03912 16121184

[B14] Horak R. E. A. Qin W. Schauer A. J. Armbrust E. V. Ingalls A. E. Moffett J. W. (2013). Ammonia oxidation kinetics and temperature sensitivity of a natural marine community dominated by Archaea. *ISME J.* 7 2023–2033. 10.1038/ismej.2013.75 23657360 PMC3965308

[B15] Ichimiya T. Takeoka K. Hiramatsu K. Hirai K. Yamasaki T. Nasu M. (1996). The Influence of Azithromycin on the Biofilm Formation of *Pseudomonas aeruginosa* in vitro. *Chemotherapy* 42 186–191. 10.1159/000239440 8983885

[B16] ISO/TS 14256–1:2003 (2003). *Soil Quality-Determination of Nitrate, Nitrite and Ammonium in Field Moist Soils by Extraction with Potassium Chloride Solution.* Geneva: International Organisation for Standardisation.

[B17] Jung M. Y. Kim J. G. Damsté J. S. S. Rijpstra W. I. C. Madsen E. L. Kim S. J. (2016). A hydrophobic ammonia-oxidizing archaeon of the *Nitrosocosmicus* clade isolated from coal tar-contaminated sediment. *Environ. Microbiol. Rep.* 8 983–992. 10.1111/1758-2229.12477 27700018

[B18] Kaplan J. B. (2011). Antibiotic-induced biofilm formation. *Int. J. Artif. Organ.* 34 737–751. 10.5301/ijao.5000027 22094552

[B19] Karner M. DeLong E. F. Karl D. M. (2001). Archaeal dominance in the mesopelagic zone of the Pacific Ocean. *Nature* 409 507–510. 10.1038/35054051 11206545

[B20] Kerou M. Offre P. Valledor L. Abby S. S. Melcher M. Nagler M. (2016). Proteomics and comparative genomics of Nitrososphaera viennensis reveal the core genome and adaptations of archaeal ammonia oxidizers. *Proc. Natl. Acad. Sci. U.S.A.* 113 7937–7946. 27864514 10.1073/pnas.1601212113 PMC5150414

[B21] Kim J. G. Park S. J. Damsté J. S. S. Schouten S. Rijpstra W. I. C. Jung M. Y. (2016). Hydrogen peroxide detoxification is a key mechanism for growth of ammonia-oxidizing archaea. *Proc. Natl. Acad. Sci. U.S.A.* 113 7888–7893. 10.1073/pnas.1605501113 27339136 PMC4948306

[B22] Kits K. D. Sedlacek C. J. Lebedeva E. V. Han P. Bulaev A. Pjevac P. (2017). Kinetic analysis of a complete nitrifier reveals an oligotrophic lifestyle. *Nature* 549 269–272. 10.1038/nature23679 28847001 PMC5600814

[B23] Könneke M. Bernhard A. E. de la Torre J. Walker C. B. Waterbury J. B. Stahl D. A. (2005). Isolation of an autotrophic ammonia oxidizing marine archaeon. *Nature* 437 543–546. 10.1038/nature03911 16177789

[B24] Kowalchuk G. A. Stephen J. R. (2001). Ammonia-oxidizing bacteria: a model for molecular microbial ecology. *Annu. Rev. Microbiol.* 55 485–529. 10.1146/annurev.micro.55.1.485 11544365

[B25] Kumar S. Stecher G. Tamura K. (2016). MEGA7: molecular evolutionary genetics analysis version 7. 0 for bigger datasets. *Mol. Biol. Evol.* 33 1–11. 10.1093/molbev/msw054 27004904 PMC8210823

[B26] Kuypers M. M. M. (2017). Microbiology: a fight for scraps of ammonia. *Nature* 549 162–163. 10.1038/549162a 28905910

[B27] Lehtovirta L. E. Prosser J. I. Nicol G. W. (2009). Soil pH regulates the abundance and diversity of Group 1.1c *Crenarchaeota*. *FEMS Microbiol. Ecol.* 70 367–376. 10.1111/j.1574-6941.2009.00748.x 19732147

[B28] Lehtovirta-Morley L. E. (2018). Ammonia oxidation: ecology, physiology, biochemistry and why they must all come together. *FEMS Microbiol. Lett.* 365:fny058. 10.1093/femsle/fny058 29668934

[B29] Lehtovirta-Morley L. E. Ross J. Hink L. Weber E. B. Gubry-Rangin C. Thion C. (2016). Isolation of “Candidatus Nitrosocosmicus franklandus”, a novel ureolytic soil archaeal ammonia oxidiser with tolerance to high ammonia concentration. *FEMS Microbiol. Ecol.* 92 1–10. 10.1093/femsec/fiw057 26976843 PMC4830249

[B30] Leininger S. Urich T. Schloter M. Schwark L. Qi J. Nicol G. W. (2006). Archaea predominate among ammonia-oxidizing prokaryotes in soils. *Nature* 442 806–809. 10.1038/nature04983 16915287

[B31] Martens-Habbena W. Berube P. M. Urakawa H. José R. Stahl D. A. (2009). Ammonia oxidation kinetics determine niche separation of nitrifying Archaea and Bacteria. *Nature* 461 976–979. 10.1038/nature08465 19794413

[B32] Nielsen P. H. Daims H. Lemmer H. Arslan-Alaton I. Olmez-Hanci T. (2009). *FISH Handbook for Biological Wastewater Treatment.* London: IWA Publishing.

[B33] Ochsenreiter T. Selezi D. Quaiser A. Bonchosmolovskaya L. Schleper C. (2003). Diversity and abundance of Crenarchaeota in terrestrial habitats studied by 16S RNA surveys and real time PCR. *Environ. Microbiol.* 5 787–797. 10.1046/j.1462-2920.2003.00476.x 12919414

[B34] Orell A. Fröls S. Albers S. V. (2013). Archaeal biofilms: the great unexplored. *Annu. Rev. Microbiol.* 67 337–354. 10.1146/annurev-micro-092412-155616 23808336

[B35] Persson T. Givskov M. Nielsen J. (2005). Quorum sensing inhibition: targeting chemical communication in gramnegative bacteria. *Curr. Med. Chem.* 12 3103–3115. 10.2174/092986705774933425 16375704

[B36] Pratscher J. Dumont M. G. Conrad R. (2011). Ammonia oxidation coupled to CO2 fixation by archaea and bacteria in an agricultural soil. *Proc. Natl. Acad. Sci. U.S.A.* 108 4170–4175. 10.1073/pnas.1010981108 21368116 PMC3053987

[B37] Reffuveille F. de la Fuente-NúÞnez C. Mansour S. Hancock R. E. W. (2014). A broad-spectrum antibiofilm peptide enhances antibiotic action against bacterial biofilms. *Antimicrob. Agents Chemother.* 58 5363–5371. 10.1128/AAC.03163-14 24982074 PMC4135845

[B38] Saini H. Chhibber S. Harjai K. (2015). Azithromycin and ciprofloxacin: a possible synergistic combination against *Pseudomonas aeruginosa* biofilm-associated urinary tract infections. *Int. J. Antimicrob. Agents* 45 359–367. 10.1016/j.ijantimicag.2014.11.008 25604277

[B39] Sauder L. A. Albertsen M. Engel K. Schwarz J. Nielsen P. H. Wagner M. (2017). Cultivation and characterization of *Candidatus* Nitrosocosmicus exaquare, an ammonia-oxidizing archaeon from a municipal wastewater treatment system. *ISME J.* 11 1142–1157. 10.1038/ismej.2016.192 28195581 PMC5398378

[B40] Shiozaki T. Ijichi M. Isobe K. Hashihama F. Nakamura K. Ehama M. (2016). Nitrification and its influence on biogeochemical cycles from the equatorial Pacific to the Arctic Ocean. *ISME J.* 10 2184–2197. 10.1038/ismej.2016.18 26918664 PMC4989309

[B41] Stahl D. A. de la Torre J. R. (2012). Physiology and diversity of ammonia-oxidizing archaea. *Annu. Rev. Microbiol.* 66 83–101. 10.1146/annurev-micro-092611-150128 22994489

[B42] Stieglmeier M. Klingl A. Alves R. J. E. Rittmann S. K. M. R. Melcher M. Leisch N. (2014). *Nitrososphaera viennensis* gen. nov., sp. nov., an aerobic and mesophilic, ammonia-oxidizing archaeon from soil and a member of the archaeal phylum Thaumarchaeota. *Int. J. Syst. Evol. Microbiol.* 64 2738–2752. 10.1099/ijs.0.063172-0 24907263 PMC4129164

[B43] Tago K. Okubo T. Shimomura Y. Kikuchi Y. Hori T. Nagayama A. (2015). Environmental factors shaping the community structure of ammonia-oxidizing bacteria and archaea in sugarcane field soil. *Microbes Environ.* 30 21–28. 10.1264/jsme2.ME14137 25736866 PMC4356460

[B44] Tourna M. Stieglmeier M. Spang A. Konneke M. Schintlmeister A. Urich T. (2011). *Nitrososphaera viennensis*, an ammonia oxidizing archaeon from soil. *Proc. Natl. Acad. Sci. U.S.A.* 108 8420–8425. 10.1073/pnas.1013488108 21525411 PMC3100973

[B45] Yamamoto R. Noiri Y. Yamaguchi M. Asahi Y. Maezono H. Ebisu S. (2015). Inhibition of polysaccharide synthesis by the sinR orthologue PGN_0088 is indirectly associated with the penetration of *Porphyromonas gingivalis* biofilms by macrolide antibiotics. *Microbiology* 161 422–429. 10.1099/mic.0.000013 25500494

[B46] Zhang C. L. Ye Q. Huang Z. Li W. Chen J. Song Z. (2008). Global occurrence of archaeal amoA genes in terrestrial hot springs. *Appl. Environ. Microbiol.* 74 6417–6426. 10.1128/AEM.00843-08 18676703 PMC2570307

