# Supplementary material for: A Two-Step Strategy for the Rapid Enrichment of Nitrosocosmicus-Like Ammonia-Oxidizing Thaumarchaea
Source: Front Microbiol. 2019 Apr 24;10:875. doi: 10.3389/fmicb.2019.00875 (PMC6491936; doi:10.3389/fmicb.2019.00875)
Supplement: Supplementary file 1 [file Table_1.DOCX]

**Frontiers in Microbiology**

**A two-step strategy for the rapid enrichment of** ***Nitrosocosmicus*-like ammonia-oxidizing thaumarchaea**

Liangting Liu, Surong Li, Jiamin Han, Weitie Lin*, Jianfei Luo*

Guangdong Key Laboratory of Fermentation and Enzyme Engineering, School of Biology and Biological Engineering, South China University of Technology, Guangzhou 510006, P R China

Correspondence：

Weitie Lin, lfwtlin@scut.edu.cn

Jianfei Luo, ljfjf2002@scut.edu.cn

MATERIALS AND METHODS

Soil samples collection and chemical analysis

Five soil samples (HN, NKY, DC, SS, and JK) were collected from Guangzhou by using a handled steel soil sampler in April 2016 (Table S1). For each site, soils were obtained by randomly collecting approximate 9 cores (with 5 cm diameter) from the top 10 cm, mixed together, and considered as one soil sample. After air dry, the soil samples were grounded into powder and sieved through a 2 mm sieve. The sieved soils were stored at –80 ^o^C until extraction of the total genomic DNA and used to determine the concentrations of total nitrogen and ammonium.

Total nitrogen concentration was determined by Kjeldahl method (Nelson and Sommers, 1980); nitrite and ammonium concentrations were determined using Griess-Ilosvay method and indophenol blue method ( ISO/TS 14256–1:2003), respectively.

**Preparation of** **Fe-EDTA solution**

196.896 g of EDTA was mixed with 800 mL of distilled water, and then the pH was adjusted to 7.4 with NaOH solid until the EDTA was completely dissolved. Then add 49.14g FeSO_4_·7H_2_O, adjust the pH to 7.4 after the dissolution, and finally add distilled water to 1 L.

Genomic DNA extraction

Before the genomic DNA extraction, the soil sample was pretreated. In brief, 0.5 g soil was placed in a 2 mL microcentrifuge tube; 0.5 mL CTAB extraction buffer (10% CTAB, 0.7 M NaCl, 240 mM potassium phosphate buffer, pH 8.0) and 0.5 mL phenol-chloroform-isoamyl alcohol solution (25:24:1, pH 8.0) were added into the tube and mixed with soil for 5 min by using Vortex Adapter (13000-V1-24, QIAGEN, Germany). After the pretreatment, genomic DNA was extracted according to a protocol that was previously reported by Griffiths (Griffiths et al., 2000). After the DNA extraction, 20 µg glycogen (Thermo Scientific, USA) was used as co-precipitant to deposit DNA from the DNA precipitated solution (30% polyethelene glycol 6000, 1.6 M NaCl). The DNA purity was determined by using a UV spectrophotometer (NanoDrop 2000, Thermo Scientific, USA) and by agarose gel electrophoresis. The DNA samples were stored at -20 ^o^C for further PCR amplification.

PCR amplification and High-throughput sequencing

Archaeal 16S rRNA and *amoA* genes were amplified using primer pairs Arch349F/Arch806R and amoAF2/amoAR (Table S3), respectively. A unique 8 bp barcode was added at the 5’ end of each forward primer. PCRs were performed using TransStart Fast Pfu Fly DNA Polymerase (Transgen, Beijing, China), with the following protocol: 95 °C for 2 min; 30 cycles consisting of 95 °C for 20 s, 53 °C for 20 s and 72 °C for 30 s; and final extension at 72 °C for 5 min. The PCR products were purified by using TaKaRa Agarose Gel DNA Purification Kit ver. 4.0 (TaKaRa, Dalian, China) and quantified by using a spectrophotometer (NanoDrop 2000, Thermo Scientific, USA). Sequencing was performed on a MiSeq 300 sequencer (Illumina, San Diego, USA) by IGE Biotechnology (Guangzhou, China). Sequence data are available in the sequence read archive of the NCBI under accession numbers SRP135839 for archaeal 16S rRNA gene and SRP135730 for archaeal *amoA* gene.

Raw data were trimmed for controlling sequence quality by Trimmomatic software (Bolger et al., 2014). Clean data were obtained after filtering reads containing ambiguities and mismatches with specific primers or those with average quality values less than 20. Data were processed using Mothur (Schloss et al., 2009) and QIIME (Caporaso et al., 2010). Chimera sequences were removed using Uchime algorithm and the operational taxonomic units (OTUs) were clusetered at the 97% nucleotide sequence identity. The taxonomic classification of 16S rRNA gene and *amoA* gene was determined on the basis of the Nucleotide collection database (nr/nt, exclude uncultured/environmental sample sequences) by blast. Alpha diversity indices including the Chaol, Shannon, and Simpson for the sampling sites were calculated and compared using the Mothur program.

**REFERENCES**

Abby, S. S., Melcher, M., Kerou, M., Krupovic, M., Stieglmeier, M., Rossel, C., et al. (2018). *Candidatus* Nitrosocaldus cavascurensis, an ammonia oxidizing, extremely thermophilic archaeon with a highly mobile genome. *Front. Microbiol.* 9, 28.

Amann, R. I., Ludwig, W., Schleifer, K. H. (1995). Phylogenetic identification and in situ detection of individual microbial cells without cultivation. *Microbiol. Rev.* 59, 143–169.

Bayer, B., Vojvoda, J., Offre, P., Alves, R. J. E., Elisabeth, N. H., Garcia, J. AL, et al. (2015). Physiological and genomic characterization of two novel marine thaumarchaeal strains indicates niche differentiation. ISME J. 10, 1–13.

Blainey, P. C., Mosier, A. C., Potanina, A., Francis, C. A., and Quake, S. R. (2011). Genome of a low-salinity ammonia-oxidizing archaeon determined by single-cell and metagenomic analysis. PLoS One 6, e16626.

Bolger, A. M., Lohse, M., and Usadel, B. (2014). Trimmomatic: A flexible trimmer for Illumina sequence data. *Bioinformatics* 30, 2114–2120.

Caporaso, J. G., Kuczynski, J., Stombaugh, J., Bittinger, K., Bushman, F. D., Costello, E. K., et al. (2010). QIIME allows analysis of high-throughput comunity sequencing data. *Nat. Methods.* 7, 335–336.

Chen, H., Yue, Y., Jin, W., Zhou, X., Wang, Q., Gao, S, et al. (2017). Enrichment and characteristics of ammonia-oxidizing archaea in wastewater treatment process. *Chem. Eng. J.* 323, 465–472.

Daebeler, A., Herbold, C. W., Vierheilig, J., Sedlacek, C. J., Pjevac, P., Albertsen, M., et al. (2018). Cultivation and genomic analysis of “*Candidatus* Nitrosocaldus islandicus,” an obligately thermophilic, ammonia-oxidizing thaumarchaeon from a hot spring biofilm in Graendalur Valley, Iceland. *Front. Microbiol.* 9, 193.

de la Torre, J. R., Walker, C. B., Ingalls, A. E., Könneke, M., and Stahl, D. A. (2008). Cultivation of a thermophilic ammonia oxidizing archaeon synthesizing crenarchaeol. *Environ. Microbiol.* 10, 810–818.

DeLong, E. F. (1992). Archaea in coastal marine environments. *Proc. Natl. Acad. Sci.* 89, 5685–5689.

Elling, F. J., Könneke, M., Mußmann, M., Greve, A., and Hinrichs, K. U. (2015). Influence of temperature , pH, and salinity on membrane lipid composition and TEX 86 of marine planktonic thaumarchaeal isolates. *Geochim. Cosmochim. Acta.* 171, 238–255.

Francis, C. A., Beman, J. M., and Kuypers, M. M. (2007). New processes and players in the nitrogen cycle: the microbial ecology of anaerobic and archaeal ammonia oxidation. *Isme J.* 1, 19.

Griffiths, R. I., Whiteley, A. S., Anthony, G., Donnell, O., Bailey, M. J., and Donnell, A. G. O. (2000). Rapid method for coextraction of DNA and RNA from natural environments for analysis of ribosomal DNA- and rRNA-based microbial community composition. *Appl. Environ. Microbiol.* 66, 5488–5491.

Hatzenpichler, R., Lebedeva, E. V, Spieck, E., Stoecker, K., Richter, A., Daims, H., et al. (2008). A moderately thermophilic ammonia-oxidizing crenarchaeote from a hot spring. *Proc. Natl. Acad. Sci.* 105, 2134–2139.

ISO/TS 14256–1:2003 (2003) Soil quality-Determination of nitrate, nitrite and ammonium in field moist soils by extraction with potassium chloride solution. International Organisation for Standardisation. Geneva, Switzerland.

Jung, M. Y., Kim, J. G., Damsté, J. S. S., Rijpstra, W. I. C., Madsen, E. L., Kim, S. J., et al. (2016). A hydrophobic ammonia-oxidizing archaeon of the *Nitrosocosmicus* clade isolated from coal tar-contaminated sediment. *Environ. Microbiol. Rep.* 8, 983–992.

Jung, M. Y., Park, S. J., Kim, S. J., Kim, J. G., Damsté, J. S. S., Jeon, C. O., et al. (2014). A mesophilic, autotrophic, ammonia-oxidizing archaeon of thaumarchaeal group I.1a cultivated from a deep oligotrophic soil horizon. *Appl. Environ. Microbiol.* 80, 3645–3655.

Jung, M. Y., Park, S. J., Min, D., Kim, J. S., Rijpstra, W. I. C., Damsté, J. S. S., et al. (2011). Enrichment and characterization of an autotrophic ammonia-oxidizing archaeon of mesophilic crenarchaeal group I.1a from an agricultural soil. *Appl. Environ. Microbiol.* 77, 8635–8647.

Kim, J. G., Park, S. J., Damsté, J. S. S., Schouten, S., Rijpstra, W. I. C., Jung, M. Y., et al. (2016). Hydrogen peroxide detoxification is a key mechanism for growth of ammonia-oxidizing archaea. *Proc. Natl. Acad. Sci.* 113, 7888–7893.

Kim, J. G., Jung, M. Y., Park, S. J., Rijpstra, W. I. C., Damsté, J. S. S., Madsen, E. L., et al. (2012). Cultivation of a highly enriched ammonia-oxidizing archaeon of thaumarchaeotal group I.1b from an agricultural soil. *Environ. Microbiol.* 14, 1528–1543.

Könneke, M., Bernhard, A. E., de la Torre, J., Walker, C. B., Waterbury, J. B., and Stahl, D. A. (2005). Isolation of an autotrophic ammonia oxidizing marine archaeon. *Nature* 437, 543–546.

Lebedeva, E. V., Hatzenpichler, R., Pelletier, E., Schuster, N., Hauzmayer, S., Bulaev, A., et al. (2013). Enrichment and genome sequence of the group I.1a ammonia-oxidizing archaeon “*Ca*. Nitrosotenuis uzonensis” representing a clade globally distributed in thermal habitats. *PLoS One* 8, e80835.

Lehtovirta-Morley, L. E., Ross, J., Hink, L., Weber, E. B., Gubry-Rangin, C., Thion, C., et al. (2016). Isolation of “*Candidatus* Nitrosocosmicus franklandus”, a novel ureolytic soil archaeal ammonia oxidiser with tolerance to high ammonia concentration. *FEMS Microbiol. Ecol.* 92, 1–10.

Lehtovirta-Morley, L. E., Ge, C., Ross, J., Yao, H., Nicol, G. W., and Prosser, J. I. (2014). Characterisation of terrestrial acidophilic archaeal ammonia oxidisers and their inhibition and stimulation by organic compounds. *Fems Microbiol. Ecol.* 89, 542–552.

Lehtovirta-Morley, L. E., Stoecker, K., Vilcinskas, A., Prosser, J. I., and Nicol, G. W. (2011). Cultivation of an obligate acidophilic ammonia oxidizer from a nitrifying acid soil. *Proc. Natl. Acad. Sci.* 108, 15892–15897.

Li, Y., Ding, K., Wen, X., Zhang, B., Shen, B., and Yang, Y. (2016). A novel ammonia-oxidizing archaeon from wastewater treatment plant: Its enrichment, physiological and genomic characteristics. *Sci. Rep.* 6, 23747.

Matsutani, N., Nakagawa, T., Nakamura, K., Takahashi, R., Yoshihara, K., and Tokuyama, T. (2011). Enrichment of a Novel Marine Ammonia-Oxidizing Archaeon Obtained from Sand of an Eelgrass Zone. *Microbes Environ.* 26, 23–29.

Nelson, D. M., Cann, I. K. O., and Mackie, R. I. (2010). Response of archaeal communities in the rhizosphere of maize and soybean to elevated atmospheric CO_2_ concentrations. *PLoS One* 5, e15897.

Nelson, D. W., and Sommers, L. E. (1980). Total nitrogen analysis for soil and plant tissues. *J. Assoc. Off. Anal. Chem.* 63, 770–778.

Nicol, G. W., Leininger, S., Schleper, C., and Prosser, J. I. (2008). The influence of soil pH on the diversity, abundance and transcriptional activity of ammonia oxidizing archaea and bacteria. *Environ. Microbiol.* 10, 2966–2978.

Palatinszky, M., Herbold, C., Jehmlich, N., Pogoda, M., Han, P., Von, B. M., et al. (2015). Cyanate as an energy source for nitrifiers. *Nature* 524, 105-108.

Park, B. J., Park, S. J., Yoon, D. N., Schouten, S., Damsté, J. S. S., and Rhee, S. K. (2010). Cultivation of autotrophic ammonia-oxidizing archaea from marine sediments in coculture with sulfur-oxidizing bacteria. *Appl. Environ. Microbiol.* 76, 7575–7587.

Qin, W., Amin, S. A., Martens-Habbena, W., Walker, C. B., Urakawa, H., Devol, A. H., et al. (2014). Marine ammonia-oxidizing archaeal isolates display obligate mixotrophy and wide ecotypic variation. Proc. Natl. Acad. Sci. 111, 12504–12509.

Qin, W., Heal, K. R., Ramdasi, R., Kobelt, J. N., Martens-Habbena, W., Bertagnolli, A. D., et al. (2017). Nitrosopumilus maritimus gen. nov., sp. nov., Nitrosopumilus cobalaminigenes sp. nov., Nitrosopumilus oxyclinae sp. nov., and Nitrosopumilus ureiphilus sp. nov., four marine ammonia-oxidizing archaea of the phylum Thaumarchaeota. Int J Syst Evol Microbiol. 67: 5067-5079.

Rotthauwe, J., and Witzel, K. (1997). The ammonia monooxygenase structural gene amoA as a functional marker : Molecular fine-scale analysis of natural ammonia-oxidizing populations. *Appl. Environ. Microbiol.* 63, 4704–4712.

Santoro, A. E., Dupont, C. L., Richter, R. A., Craig, M. T., Carini, P., McIlvin, M. R., et al. (2015). Genomic and proteomic characterization of “ *Candidatus* Nitrosopelagicus brevis”: An ammonia-oxidizing archaeon from the open ocean. *Proc. Natl. Acad. Sci.* 112, 1173–1178.

Sauder, L. A., Albertsen, M., Engel, K., Schwarz, J., Nielsen, P. H., Wagner, M., et al. (2017). Cultivation and characterization of *Candidatus* Nitrosocosmicus exaquare, an ammonia-oxidizing archaeon from a municipal wastewater treatment system. *ISME J.* 11, 1142–1157.

Sauder, L. A., Engel, K., Lo, C. C., Chain, P., and Neufeld, J. D. (2018). Cultivation and characterization of *Candidatus* Nitrosotenuis aquariensis, an ammonia-oxidizing archaeon from a freshwater aquarium biofilter. *Appl. Environ. Microbiol.*

Schloss, P. D., Westcott, S. L., Ryabin, T., Hall, J. R., Hartmann, M., Hollister, E. B., et al. (2009). Introducing mothur: Open-source, platform-independent, community-supported software for describing and comparing microbial communities. *Appl. Environ. Microbiol.* 75, 7537–7541.

Suzuki, M. T., Taylor, L. T., and DeLong, E. F. (2000). Quantitative analysis of small-subunit rRNA genes in mixed microbial populations via 5 ′ - nuclease assays. *Appl. Environ. Microbiol.* 66, 4605–4614.

Takai, K., and Horikoshi, K. (2000). Rapid detection and quantification of members of the archaeal community by quantitative PCR using fluorogenic probes. *Appl. Environ. Microbiol.* 66, 5066.

Tourna, M., Freitag, T. E., Nicol, G. W., and Prosser, J. I. (2008). Growth, activity and temperature responses of ammonia-oxidizing archaea and bacteria in soil microcosms. *Environ. Microbiol.* 10, 1357–1364.

Tourna, M., Stieglmeier, M., Spang, A., Konneke, M., Schintlmeister, A., Urich, T., et al. (2011). *Nitrososphaera viennensis*, an ammonia oxidizing archaeon from soil. *Proc. Natl. Acad. Sci.* 108, 8420–8425.

Zhalnina, K. V., Dias, R., Leonard, M. T., De Quadros, P. D., Camargo, F. A. O., Drew, J. C., et al. (2014). Genome sequence of *Candidatus* nitrososphaera evergladensis from group I.1b enriched from everglades soil reveals novel genomic feature s of the ammonia-oxidizing archaea. *PLoS One* 9, 21–23.

**TABLE S1** Information of AOA isolates and enrichments

| AOA Strain | Source | Abundance^a^ | Duration^b^ | Antibiotics^c^ | Reference |
| --- | --- | --- | --- | --- | --- |
| *Nitrosopumilus maritimus* SCM1 | Tropical marine aquarium | 100% | N/A^d^ | N/A | (Könneke et al., 2005) |
| *Ca**.* Nitrososphaera gargensis | Hot spring | 50% | 6 years | N/A | (Hatzenpichler et al., 2008) |
| *Ca.* Nitrosocaldus yellowstonii HL72 | Hot spring | >90% | 2 years | N/A | (de la Torre et al., 2008) |
| *Ca.* Nitrosopumilus koreensis AR1 | Marine sediment | >80% | 2 years | Streptomycin, Kanamycin | (Park et al., 2010) |
| *Nitrososphaera viennensis* EN76 | Garden soil | 100% | 2 years | Streptomycin, Kanamycin, Ampicillin | (Tourna et al., 2011) |
| *Ca.* Nitrosotalea devanaterra Nd1 | Acid soil | 90% | N/A | Streptomycin | (Lehtovirta-Morley et al., 2011) |
| *Ca.* Nitrosoarchaeum koreensis MY1 | Agricultural soil | 90% | 2 years | Streptomycin, Kanamycin, Ampicillin, Penicillin, Gentamicin, Lincomycin | (Jung et al., 2011) |
| *Ca.* Nitrosoarchaeum limnia SFB1 | Estuarine sediment | 84% | N/A | N/A | (Blainey et al., 2011) |
| *Ca.* Nitrosopumilus sp. NM25 | Coastal sand | 89% | 2 years | N/A | (Matsutani et al., 2011) |
| *Ca.* Nitrososphaera sp. JG1 | Agricultural soil | 89% | a year | Streptomycin, Kanamycin, Ampicillin | (Kim et al., 2012) |
| *Ca.* Nitrosotenuis uzonensis N4 | Hot spring | 50% | 7 years | Streptomycin, Vancomycin | (Lebedeva et al., 2013) |
| *Ca.* Nitrososphaera evergladensis SR1 | Agricultural soil | 50% | 1 years | Gentamicin, Tetracycline, Erythromycin | (Zhalnina et al., 2014) |
| *Ca.* Nitrosotenuis chungbukensis MY2 | Agricultural soil | 91% | 3 years | Ampicillin, Penicillin | (Jung et al., 2014) |
| *Ca.* Nitrosotalea sp. Nd2 | Acid agricultural soil | 100% | 3 years | Kanamycin | (Lehtovirta-Morley et al., 2014) |
| *Nitrosopumilus cobalaminigenes* HCA1 | 50 m depth marine water | 100% | N/A | Streptomycin | (Qin et al., 2014) |
| *Nitrosopumilus ureiphilus* PS0 | Marine surface sediment | 100% | N/A |  |  |
| *Ca.* Nitrosopelagicus brevis CN25 | Natural seawater | 90-95% | N/A | Streptomycin, Ampicillin | (Santoro et al., 2015) |
| *Nitrosopumilus maritimus* NAO2 | Surface ocean water | 100% | N/A | Streptomycin, Gentamicin | (Elling et al., 2015) |
| *Nitrosopumilus maritimus* NAO6 | Surface ocean water | 100% | N/A | Streptomycin, Kanamycin |  |
| *Nitrososphaera gargensis* Ga9.2 | AOA enrichment | 100% | 16 mouth | Kanamycin, penicillin, streptomycin, carbenicillin, ampicillin, erythromycin, doxycyclin | (Palatinszky et al., 2015) |
| *Ca.* Nitrosotenuis cloacae SAT1 | Wastewater treatment plant | 91% | 1 year | Streptomycin, Ampicillin | (Li et al., 2016) |
| *Ca.* Nitrosopumilus piranensis D3C | Seawater | >99% | 2 years | Streptomycin, Kanamycin, Carbenicillin, Spectinomycin, Ofloxacin, Chloramphenicol | (Bayer et al., 2016) |
| *Ca.* Nitrosopumilus adriaticus NF5 | Seawater | >99% | 2 years |  |  |
| *Ca.* Nitrosocosmicus oleophilus MY3 | Coal tar-contaminated sediment | >99% | N/A | Ampicillin, Kanamycin, Streptomycin, Clarithromycin | (Jung et al., 2016) |
| *Nitrosopumilus maritimus* DDS1 | 200 m depth of seawater | 100% | 2 years | N/A | (Kim et al., 2016) |
| *Ca.* Nitrosocosmicus franklandus C13 | Arable soil | 100% | N/A | Ampicillin, Carbenicillin, Gentamycin, Clindamycin, Rifampicin | (Lehtovirta-Morley et al., 2016) |
| *Ca.* Nitrosocosmicus exaquare G61 | Wastewater treatment plant | 99% | 3 years | Kanamycin, Streptomycin, Ampicillin | (Sauder et al., 2017) |
| *Nitrosopumilus oxyclinae* HCE1 | 17 m depth marine water | 100% | N/A | Streptomycin | (Qin et al., 2017) |
| *Ca. Nitrososphaera* sp. OTU8 | Wastewater treatment plant | 91% | N/A | Streptomycin, Kanamycin, Ampicillin, Carbenicillin, Tetracycline | (Chen et al., 2017) |
| *Ca.* Nitrosocaldus islandicus | Hot spring biofilm | 85% | N/A | Spiramycin | (Daebeler et al., 2018) |
| *Ca.* Nitrosocaldus cavascurensis | Hot spring mud | 92% | 4 years | Sulfamethazine, Rifampicin, Novobiocin | (Abby et al., 2018) |
| *Ca.* Nitrosotenuis aquariensis | Freshwater aquarium biofilter | 97-99% | N/A | Streptomycin, Ampicillin, Nalidixic acid | (Sauder et al., 2018) |

Abundance^a^: the proportion of AOA in the enrichment; Duration^b^: the duration of time to obtain high purity AOA; Antibiotics^c^: antibiotics used in the enrichment experiment; N/A^d^: no report in the related reference.

**TABLE S2** Characteristics of the soil samples

| Sample | Geographical coordinates | Source | Total N(g/kg) | NH_4_^+^-N (mg/kg) | pH |
| --- | --- | --- | --- | --- | --- |
| HN | N23°10′3.05″, E113°21′39.71″ | Paddy soil | 1.39±0.21 | 51.6±2.8 | 6.60±0.09 |
| NKY | N23°06′8.63″, E113°20′17.92″ | Vegetable soil | 1.14±0.24 | 16.4±1.5 | 5.07±0.08 |
| DC | N22°52′53.43″, E113°28′35.3″ | Vegetable soil | 0.73±0.12 | 13.2±1.5 | 5.32±0.06 |
| SS | N23°03′8.29″, E113°24′20.44″ | Vegetable soil | 0.89±0.09 | 25.5±2.1 | 5.72±0.03 |
| JK | N23°07′1.54″, E113°12′15.26″ | Garden soil | 1.43±0.36 | 8.4±0.4 | 6.42±0.04 |
| HN_SD | N23°10′4.33″, E113°21′46.89″ | Paddy soil | 1.89±0.17 | 66.1±1.8. | 6.71±0.05 |
| HN_BJ | N23°09′29.38″, E113°22′45.21″ | Banana soil | 1.56±0.07 | 58.7±0.8 | 6.34±0.04 |

TABLE S3 Alpha diversity of high-throughput sequencing

| Gene | Sample | No.of reads | OTUs | Chaol | Simpson | Shannon |
| --- | --- | --- | --- | --- | --- | --- |
| 16S rRNA | HN | 7101 | 501 | 596.7984 | 0.944780 | 5.956135 |
|  | NKY | 8038 | 698 | 863.9034 | 0.945942 | 6.159469 |
|  | DC | 5251 | 525 | 642.7660 | 0.943757 | 6.311467 |
|  | SS | 5130 | 291 | 422.0476 | 0.757881 | 6.311467 |
|  | JK | 18204 | 893 | 848.8808 | 0.907438 | 6.181019 |
| *amoA* | HN | 3307 | 371 | 484.9706 | 0.895281 | 5.519664 |
|  | NKY | 10138 | 656 | 648.2901 | 0.895281 | 5.276606 |
|  | DC | 6139 | 476 | 571.7394 | 0.79264 | 4.788574 |
|  | SS | 8043 | 612 | 677.7884 | 0.952136 | 5.897822 |
|  | JK | 10286 | 654 | 631.145 | 0.868085 | 5.531379 |

TABLE S4 Primers and probes used in this study

| Application | Target | Primer/Probe | Sequence(5’-3’) | Reference |
| --- | --- | --- | --- | --- |
| High-throughput sequencing | Archaeal 16S rRNA gene | Arch349F | GYG CAS CAG KCG MGA AW | (Takai et al., 2000) |
|  |  | Arch806R | GGA CTA CVS GGG TAT CTA AT | (Takai et al., 2000) |
|  | Archaeal *amoA* | amoAF2 | GGT NGC VAA RRG HGC WTG G | (Nelson et al., 2010) |
|  |  | amoAR | GCG GCC ATC CAT CTG TAT GT | (Francis et al., 2005) |
| Clone library | Archaeal 16S rRNA gene | 21F | TTC CGG TTG ATC CYG CCG GA | (DeLong, 1992) |
|  | Universal 16S rRNA gene | 1492R | GYY ACC TTG TTA CGA CTT | (Nicol et al., 2008) |
|  | Archaeal *amoA* | CrenamoA23f | ATG GTC TGG CTW AGA CGC | (Tourna et al., 2008) |
|  |  | CrenamoA616r | GCC ATC CAT CTG TAT GTC CA | (Tourna et al., 2008) |
| Ammonia-oxidizing bacteria detection | Bacterial *amoA* | amoA-1F | GGG GTT TCT ACT GGT GGT | (Rotthauwe and Witzel, 1997) |
|  |  | amoA-2R | CCC CTC KGS AAA GCC TTC TTC | (Rotthauwe and Witzel, 1997) |
| Quantiﬁcation | Thaumarchaeal 16S rRNA | SS16S-1F | GCG CGA AAC CTC TGC AAT AG | This study |
|  |  | SS16S-1R | CCC AAT AAA CGT CCC GAC CA | This study |
|  | Bacterial 16S rRNA | 1369F | CGG TGA ATA CGT TCY CGG | (Suzuki et al., 2000) |
|  |  | 1492R | GYY ACC TTG TTA CGA CTT | (Nicol et al., 2008) |
| FISH | Archaeal 16S rRNA | ARCH915 | GTG CTC CCC CGC CAA TTC CT | (Amann et al., 1995) |
|  | Bacterial 16S rRNA | EUB338 | GCT GCC TCC CGT AGG AGT | (Amann et al., 1995) |

TABLE S5 Nucleotide sequence accession number

| **Strain or soil sample name** | **Gene** | **Amplicon raw data** | **Accession number** |
| --- | --- | --- | --- |
| *Ca.* Nitrosocosmicus sp. SS | 16S rRNA | - | MH032835 |
| *Ca.* Nitrosocosmicus sp. SS | amoA | - | MH024494 |
| *Ca.* Nitrosocosmicus sp. HNSD | 16S rRNA | - | MK393911 |
| *Ca.* Nitrosocosmicus sp. HNSD | amoA | - | MK396101 |
| *Ca.* Nitrosocosmicus sp. HNBJ | 16S rRNA | - | MK393915 |
| *Ca.* Nitrosocosmicus sp. HNBJ | amoA | - | MK396102 |
| HN, NKY, DC, SS, JK | - | archaeal 16S rRNA | SRR6846294 |
| HN, NKY, DC, SS, JK | - | archaeal amoA | SRR6837777 |

**a**

**b**

**b**

FIGURE S1 Relative abundance as a percentage of archaeal 16S rRNA (a) and *amoA* (b) gene at the genus level in five soil sampling sites HN, NKY, DC, SS and JK.

**FIGURE S2** Nitrite accumulation after consumed 14mg/L NH_4_-N in the AOA enrichments with different antibiotic combinations. S: streptomycin, K: kanamycin, A: ampicillin, C: carbenicillin, T: tetracycline, 50 mg/L of each antibiotic was added before inoculation. Error bars indicate the standard error of the mean for biological triplicates.


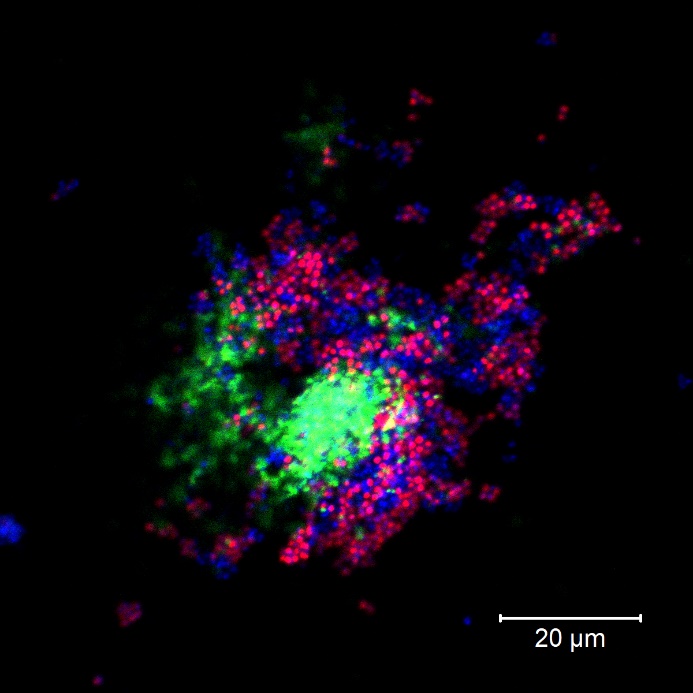

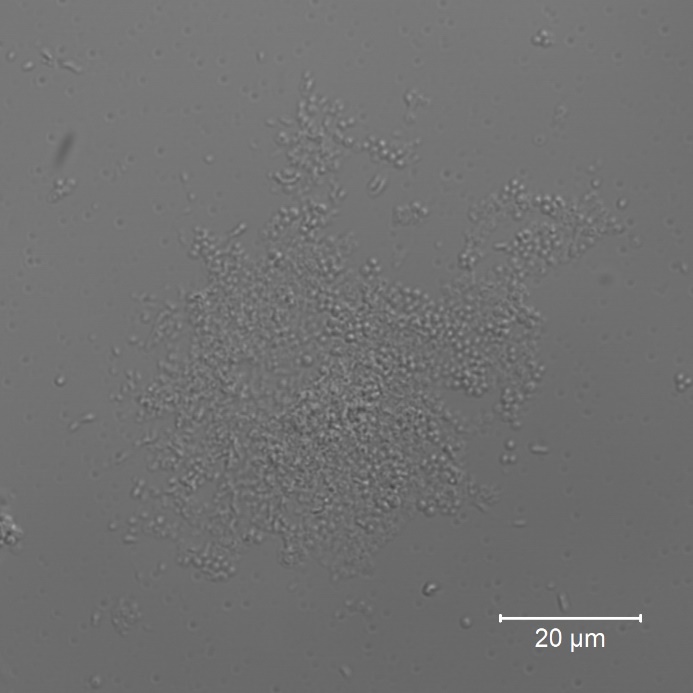


**a**

**b**

**
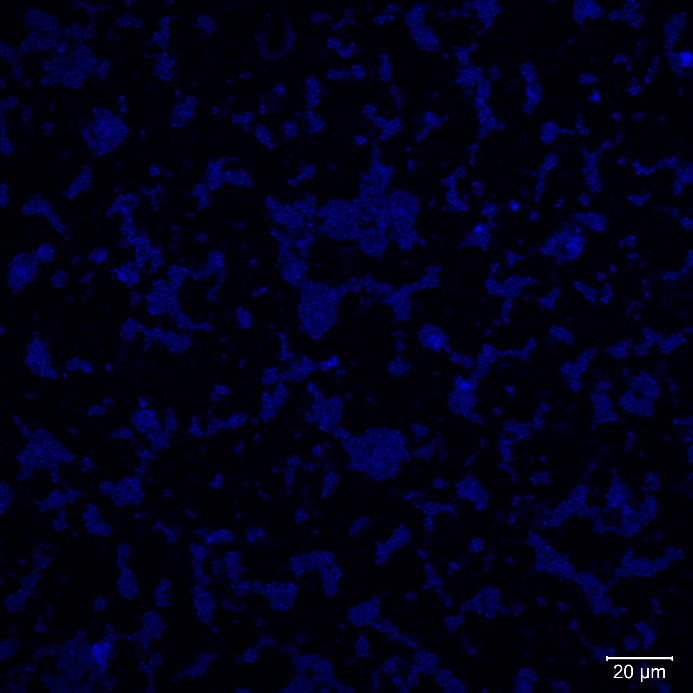
**
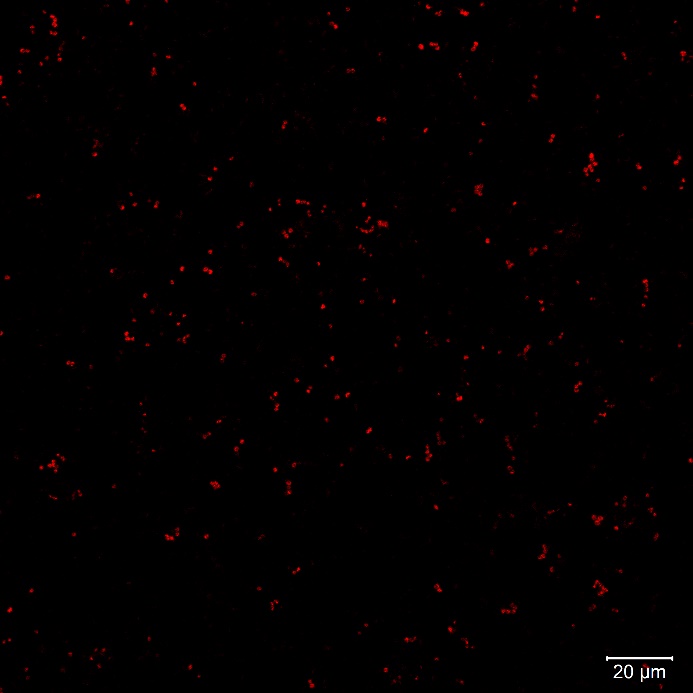


**c**


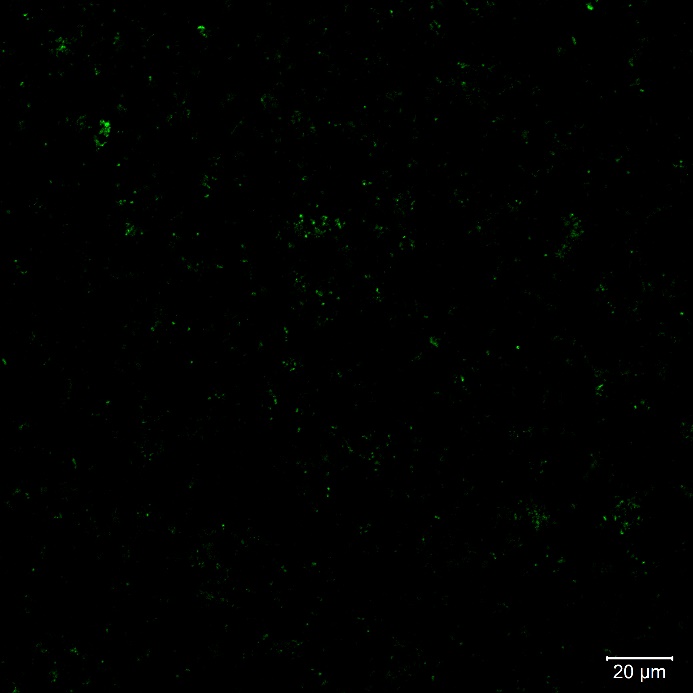

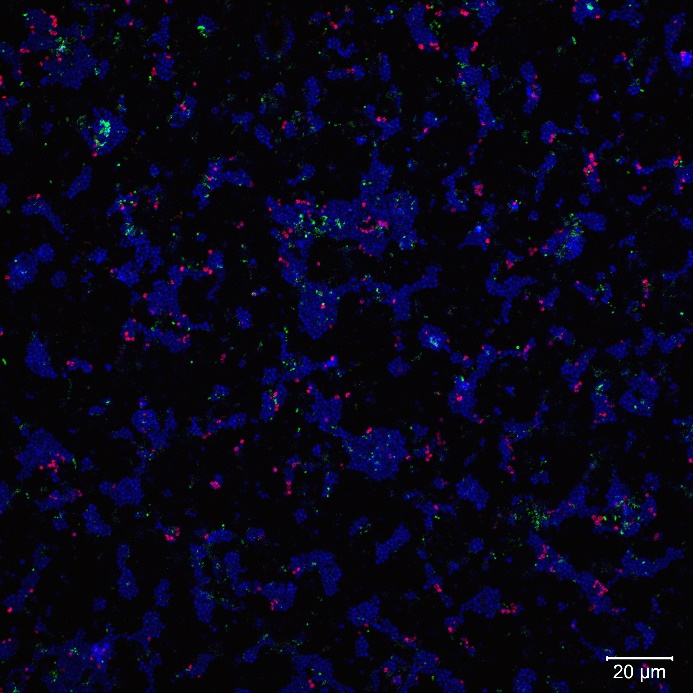


**
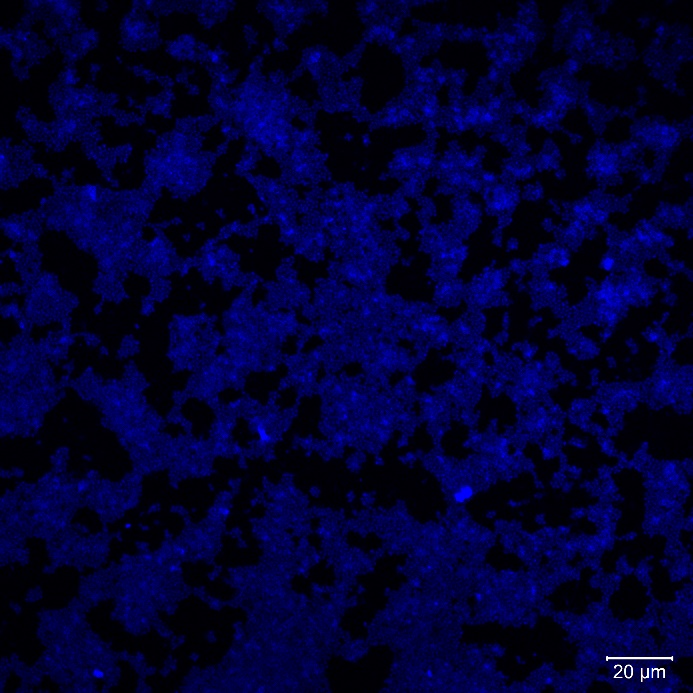

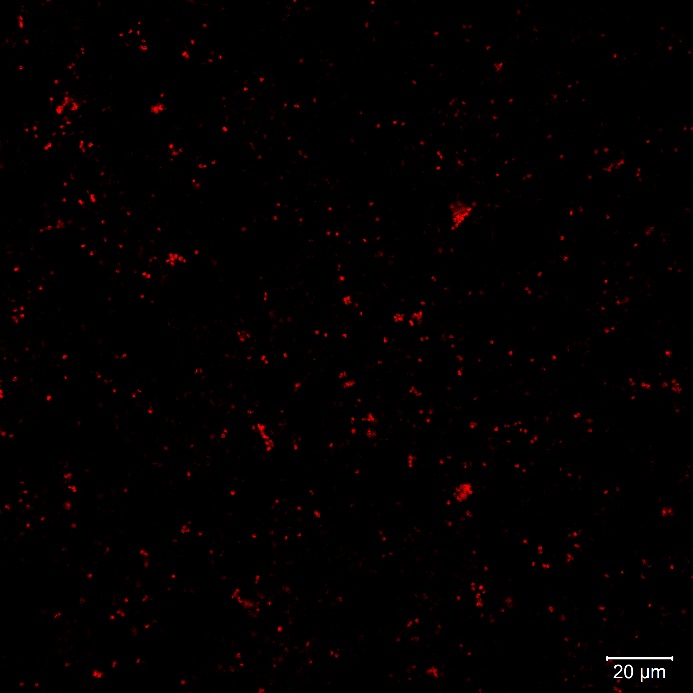
**

**d**

**
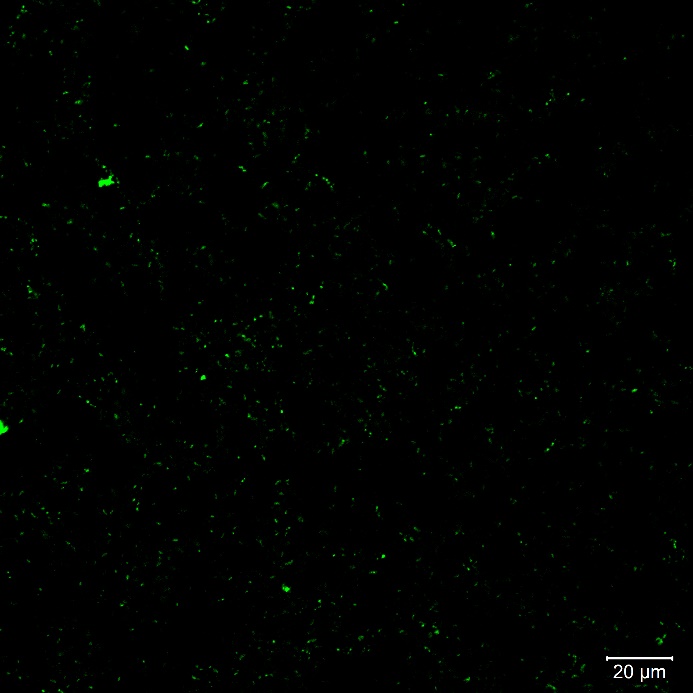

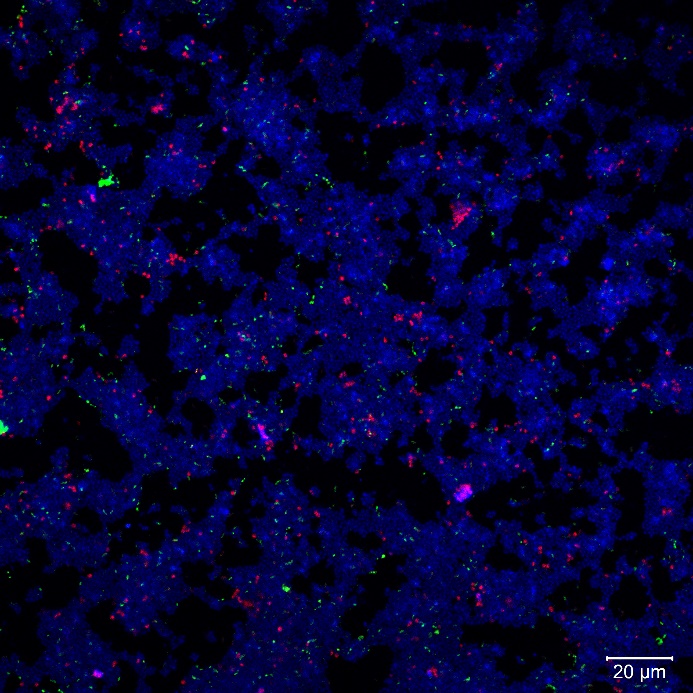
**

**
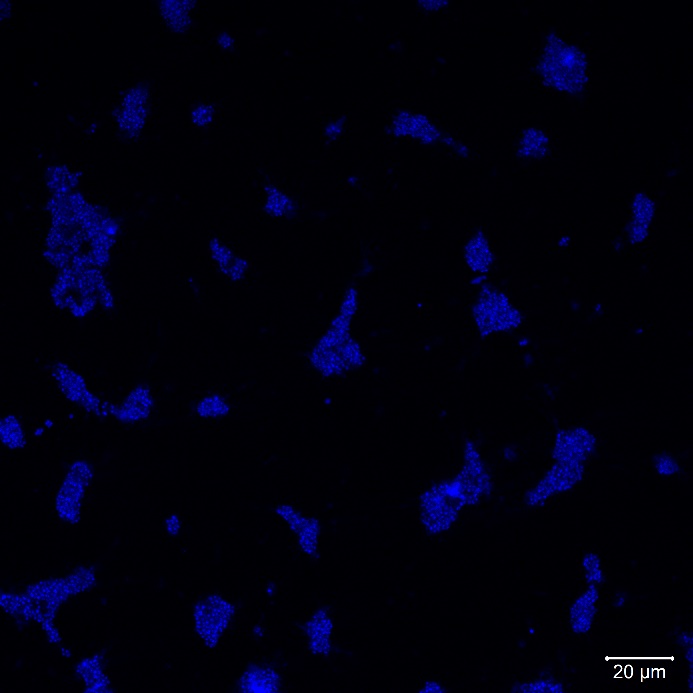

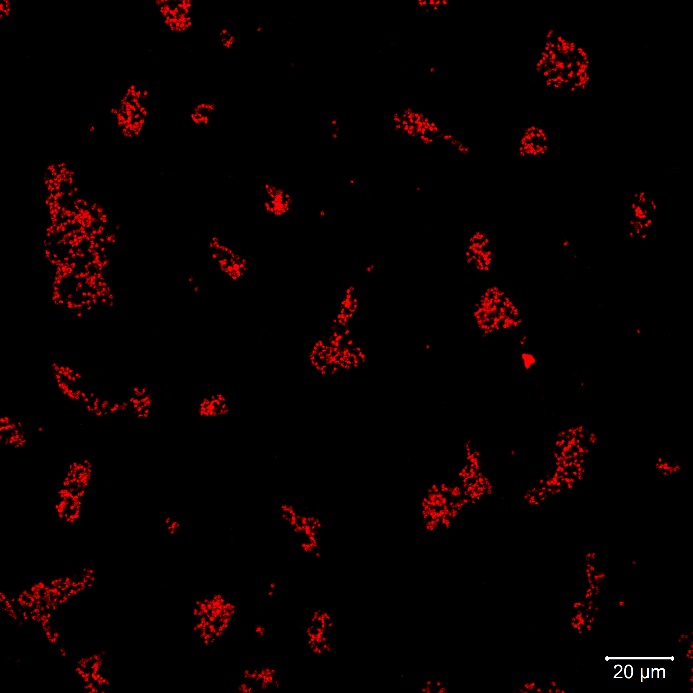
**

**e**

**
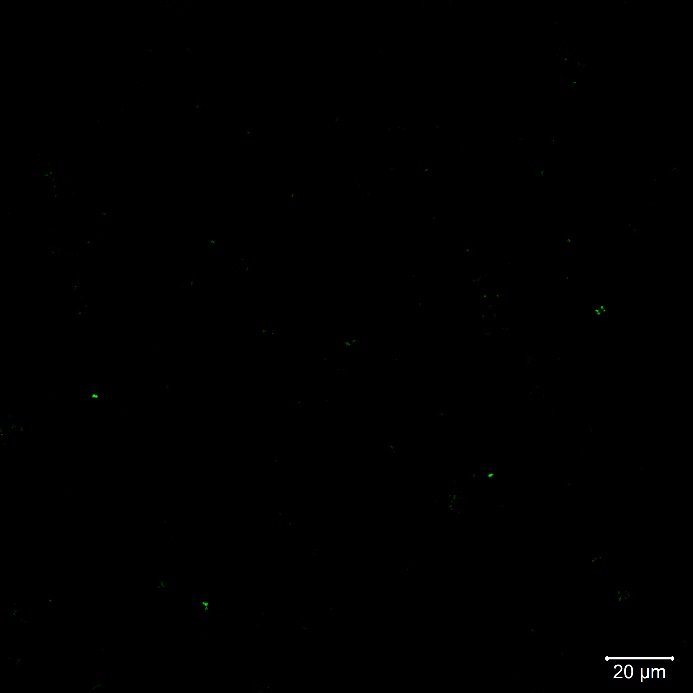

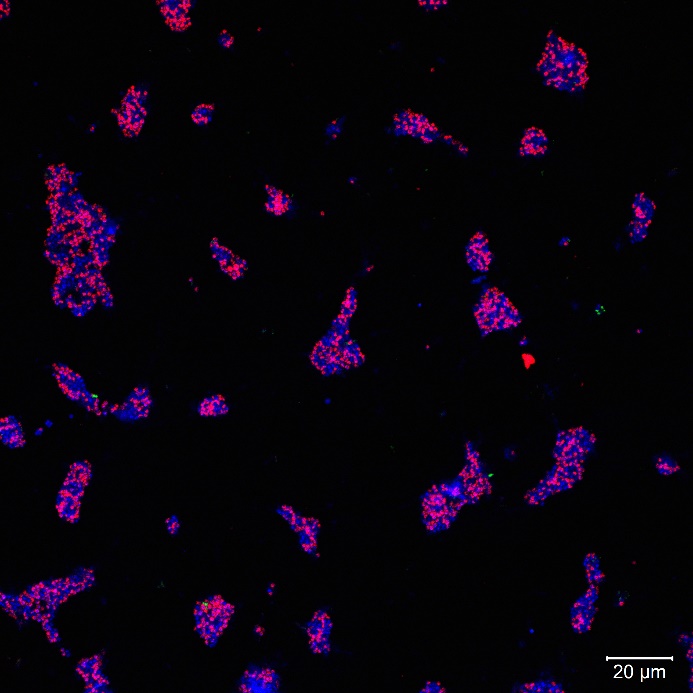
**

**
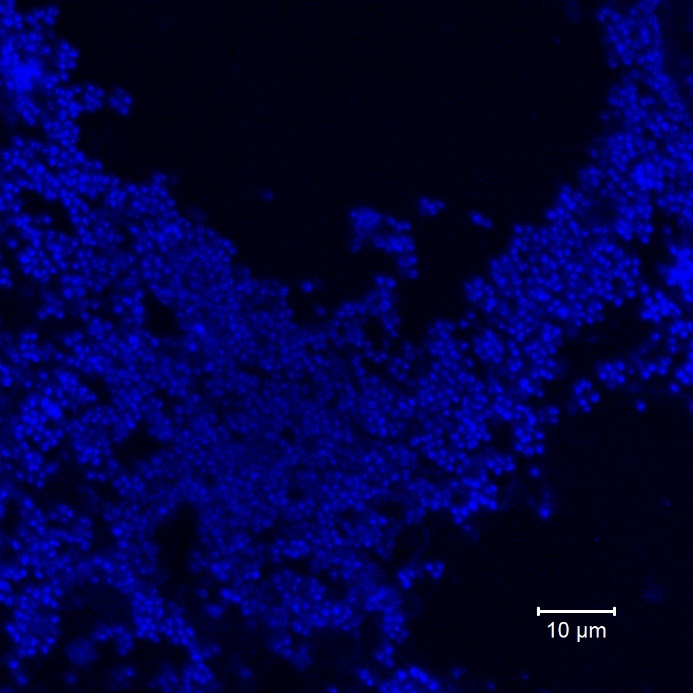

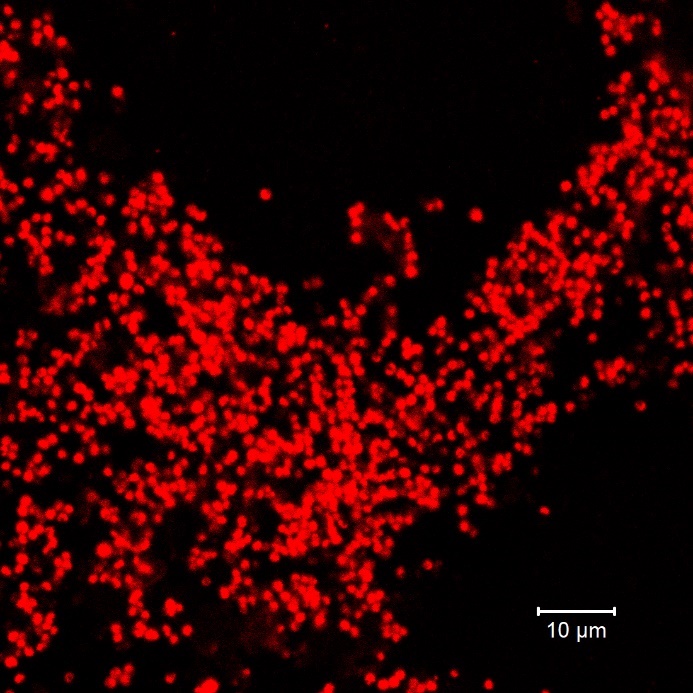

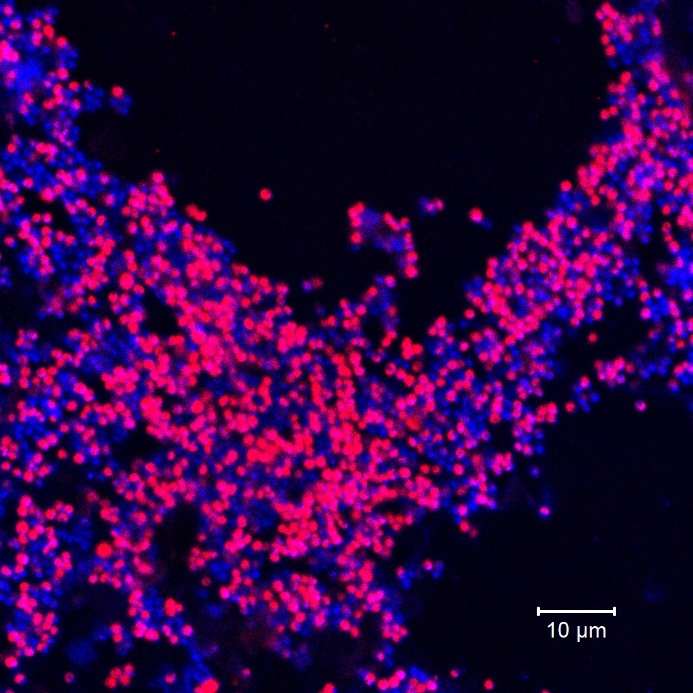
**

**f**


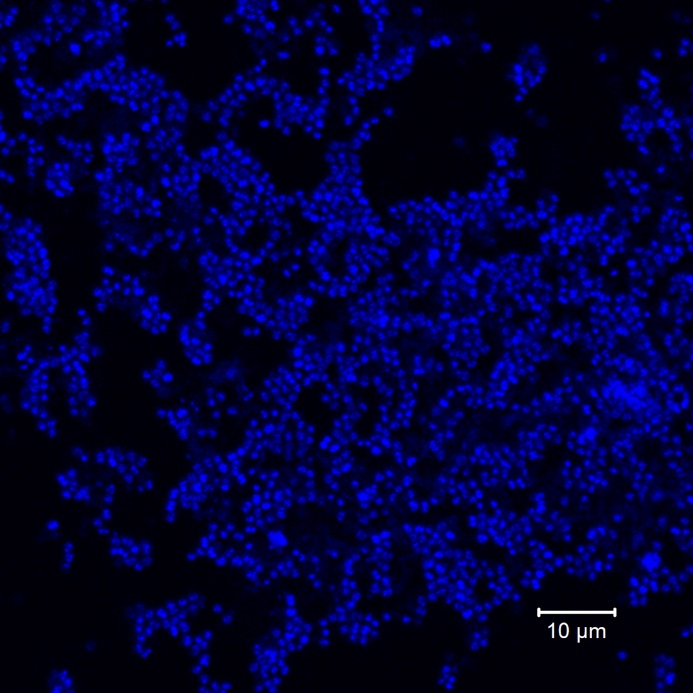

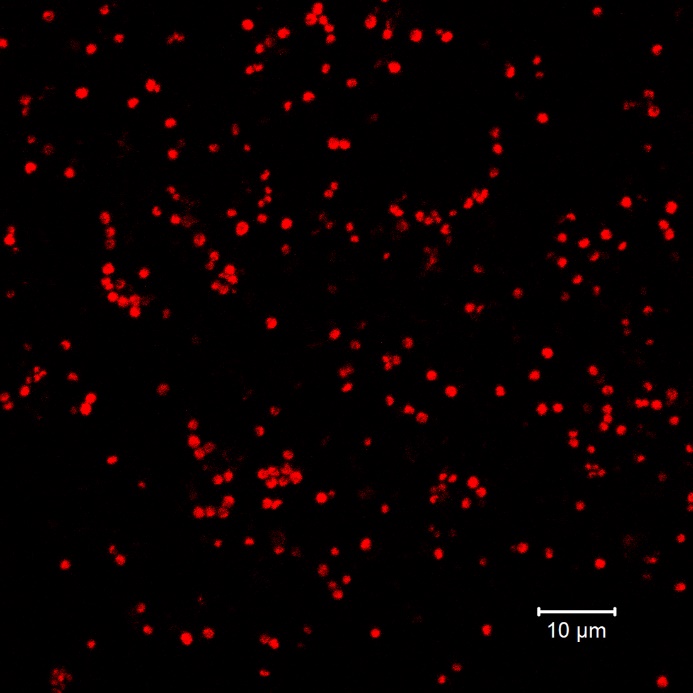

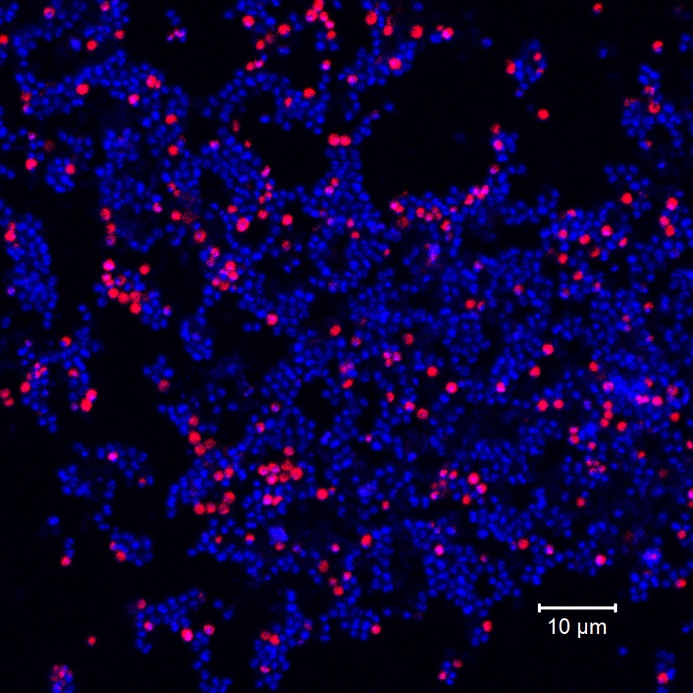


**g**

**FIGURE S3** FISH (a) and DIC (b) profiles of biofilms collected from quartz sand of the AOA enrichments (treated with kanamycin and ampicillin). The FISH split layers of the biofilms with no antibiotics(c), kanamycin and ampicillin (d) and ciprofloxacin and azithromycin (e). The FISH split layers of the dispersed cell with ciprofloxacin and azithromycin (f) and kanamycin and ampicillin (g). Objective: Plan-Apochromat 40x/1.3 Oil DIC M27, red: Alexa Fluor 546 labeled archaeal 16S rRNA, green: Alexa Fluor 488 labeled bacterial 16S rRNA, blue: DAPI.


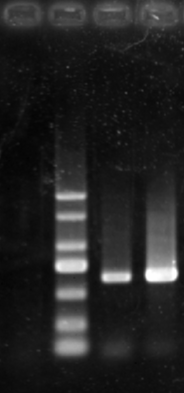


Marker SD BJ

**c**

**b**

**a**

2000

1500

1000

750

500

250

100


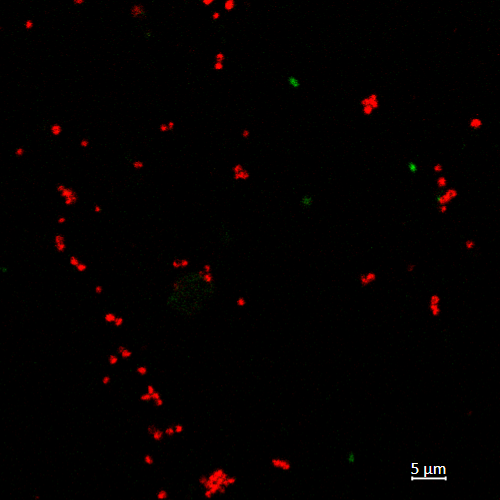

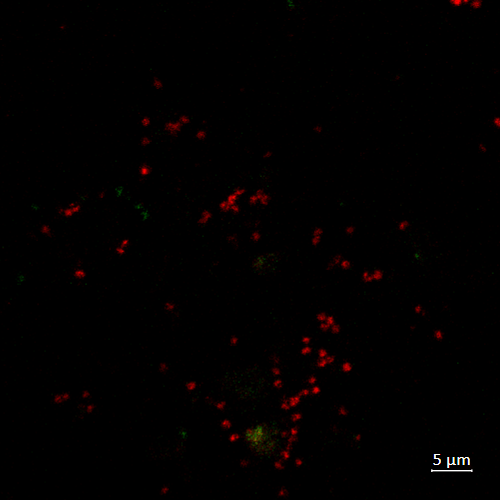


**f**

**e**

**d**

**FIGURE S4** (a) PCR detection of archaeal amoA (primer: crenamoA23f/crenamoA616r) on new soil samples.SD, HN_SD sample. BJ, HN_BJ sample. Profiles of the first stage of enrichment (using quartz sand, treated with ampicillin and kanamycin) for HN_SD (b) and HN_BJ (c) samples. Profiles of the second stage of enrichment (treated with azithromycin and ciprofloxacin) for HN_SD and HN_BJ (d) samples. FISH profiles of HN_SD (e) and HN_BJ (f) enrichment purified by azithromycin and ciprofloxacin. Objective: Plan-Apochromat 40x/1.3 Oil DIC M27, red: Alexa Fluor 546 labeled archaeal 16S rRNA, green: Alexa Fluor 488 labeled bacterial 16S rRNA. Error bars indicate the standard error of the mean for technical triplicates.

**FIGURE S5** Nitrite produced by AOA when in presence of different concentrations of pyruvate. Error bars indicate the standard error of the mean for biological triplicates.
